# Supplementary material for: Morphology-Aware Peptide Discovery via Masked Conditional Generative Modeling
Source: ACS Nano. 2026 Apr 21;20(17):13323–38. doi: 10.1021/acsnano.6c02811 (PMC13151066; doi:10.1021/acsnano.6c02811)
Supplement: Supplementary file 1 [file nn6c02811_si_001.pdf]

Morphology-Aware Peptide Discovery via Masked Conditional Generative Modeling

Nuno Costa[†] and Julija Zavadlav[*,†,‡]

†*Multiscale Modeling of Fluid Materials, Department of Engineering Physics and Computation, TUM School of Engineering and Design, Technical University of Munich, 85748 Garching, Germany*

‡*Atomistic Modeling Center, Munich Data Science Institute, Technical University of Munich, 85748 Garching, Germany*

E-mail: julija.zavadlav@tum.de

Supplementary Material

Dataset Analysis

Table 1: **Aggregation-propensity differences for overlapping peptides.** Summary statistics of aggregation-propensity differences for the 56 peptides present in both source datasets. We report $\Delta\mathrm{AP} = \mathrm{AP}_{\mathrm{Teijlingen\&Tuttle}} - \mathrm{AP}_{\mathrm{Wang}}$.

Count	Mean	Std. dev.	Min	25% quantile	Median	75% quantile	Max
56	−0.090	0.137	−0.395	−0.180	−0.095	0.013	0.176

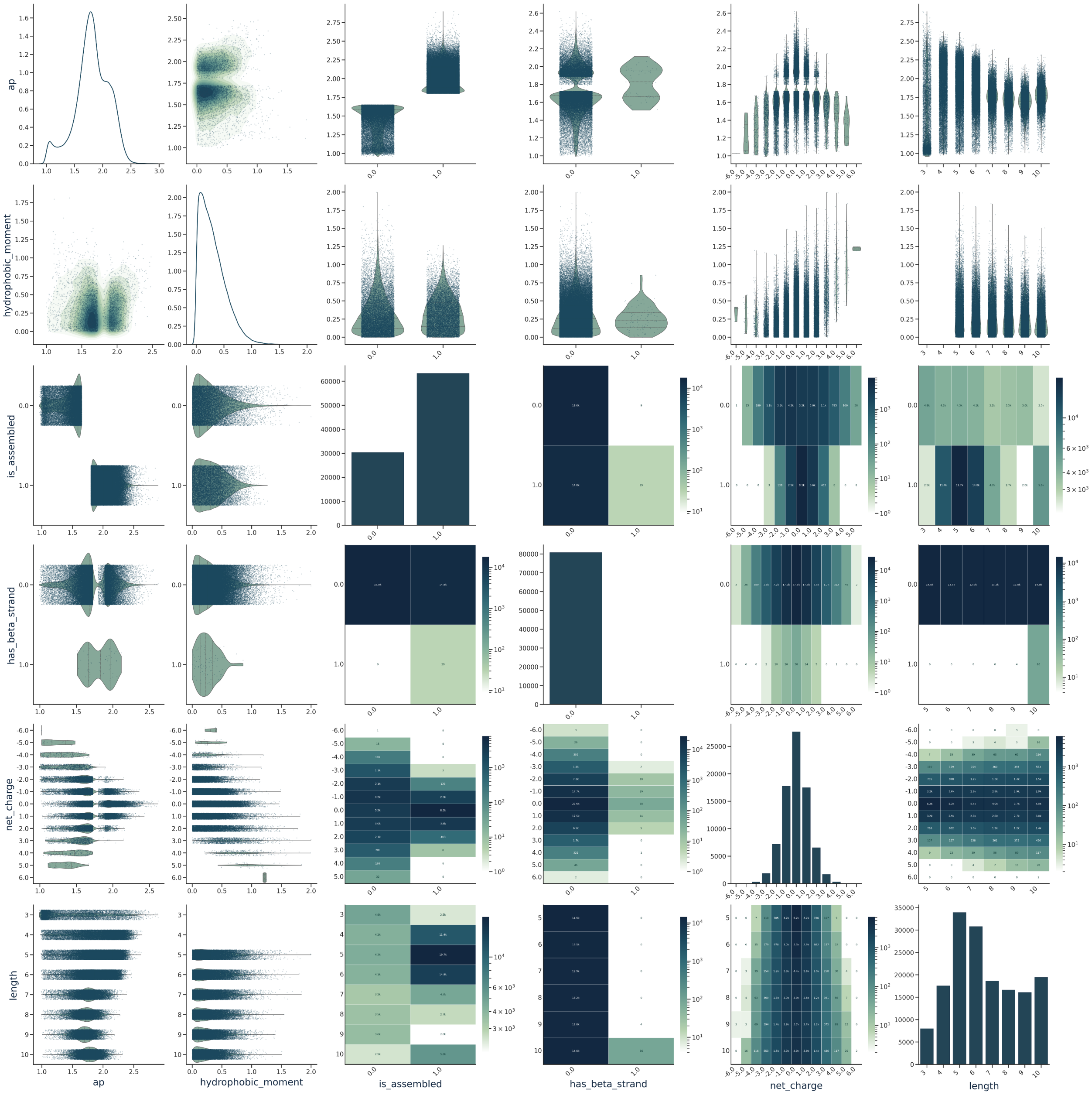

Figure 1: **Pairwise relationships among PepMorph descriptors.** Diagonal panels show marginal distributions, off-diagonal panels show scatter–density plots, and heatmaps summarize joint distributions of discrete/binary descriptors, with darker shades indicating higher counts.

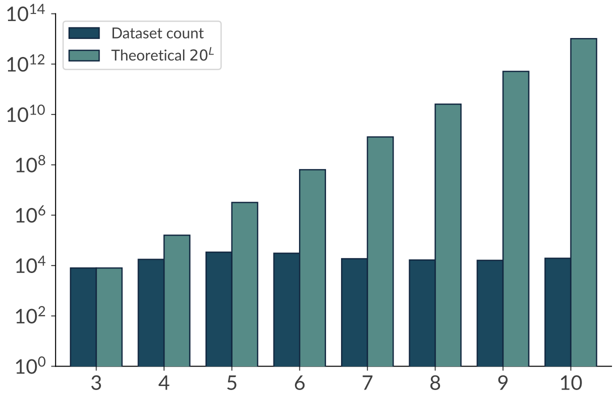

Figure 2: **PepMorph coverage versus theoretical sequence space.** Number of dataset entries per peptide length compared with the theoretical sequence space (20^L). While the dataset fully covers all length–3 peptides, coverage decreases rapidly with length as the combinatorial space grows exponentially.

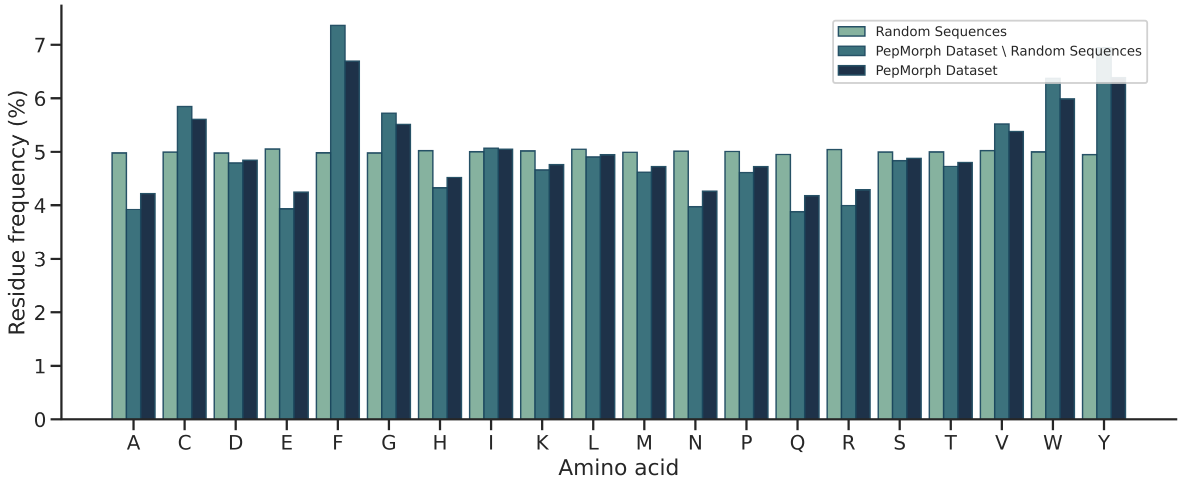

Figure 3: **Amino-acid composition shift induced by introducing the random peptides.** Residue-frequency distributions for the synthetic random cohort (left bars), the PepMorph dataset excluding random sequences (middle bars), and the final, merged PepMorph dataset (right bars). The random sequences are approximately uniform across the 20 canonical amino acids (around 5% each), consistent with uniform residue sampling. In contrast, the original dataset exhibits a strong compositional bias, with over-representation of residues C, F, W and Y. After merging, this bias is attenuated, yielding a more balanced amino-acid distribution.

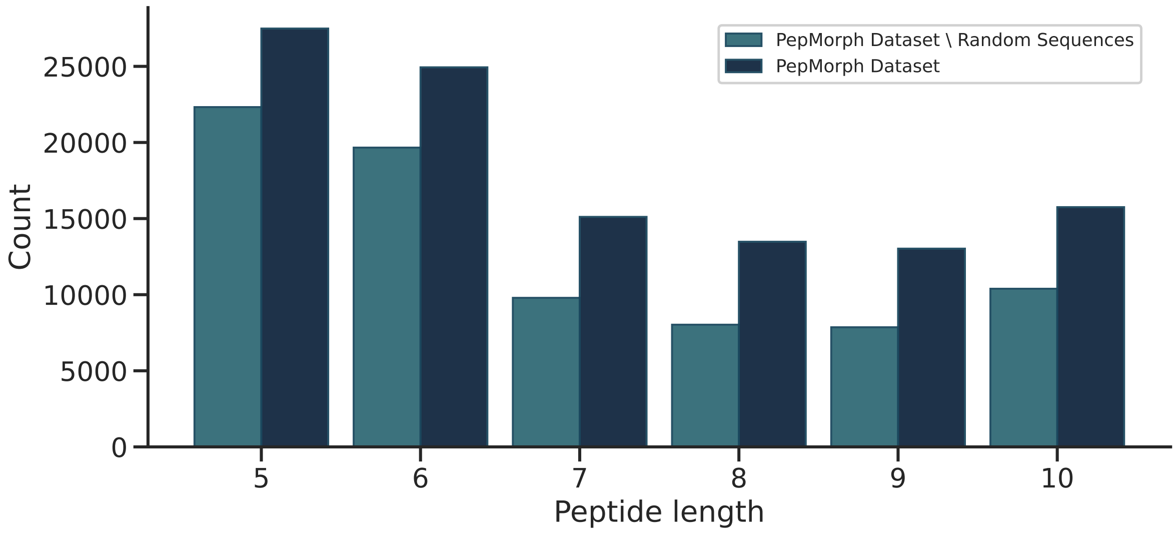

Figure 4: **Length-distribution shift induced by introducing the random peptides.** Counts of peptides by sequence length for the PepMorph dataset excluding the synthetic random cohort (left bars) and for the final, merged PepMorph dataset (right bars). Adding the synthetic cohort increases the number of available sequences at each length while largely preserving the overall length profile of the original dataset.

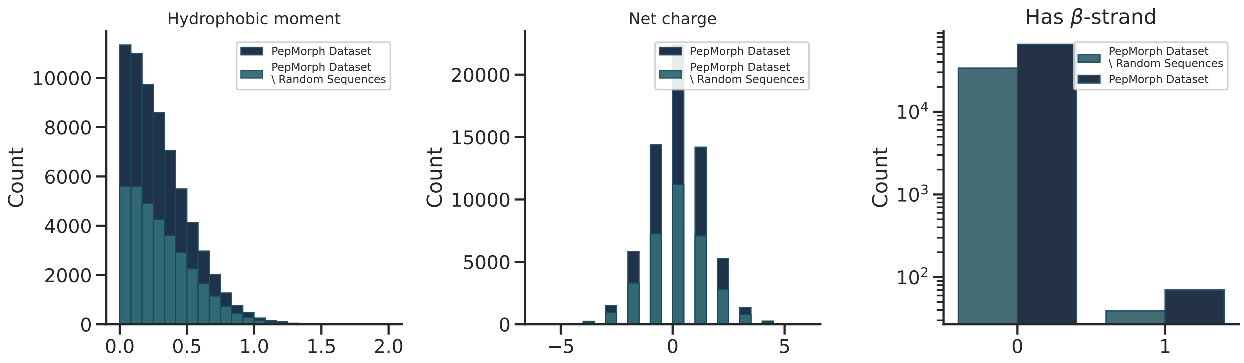

Figure 5: **Effect of synthetic augmentation on proxy-descriptor distributions.** Marginal distributions of the monomer-level proxy descriptors used for morphology steering: hydrophobic moment (left), net charge (middle), and the β-strand flag (right). In each panel, we compare the PepMorph dataset excluding synthetic random sequences (lighter bars) against the final, merged PepMorph dataset (darker bars). The synthetic cohort increases coverage across the proxy-descriptor space while preserving the qualitative shape of the distributions.

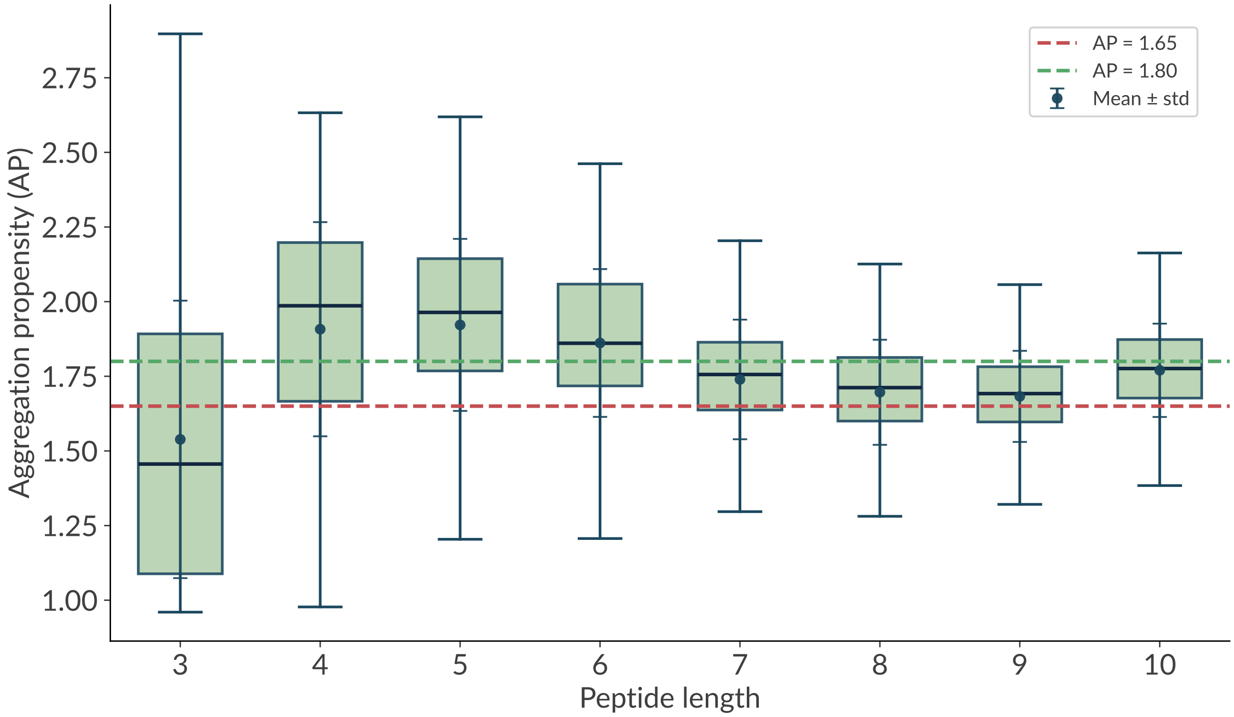

Figure 6: **Length-stratified aggregation-propensity distribution.** Boxes indicate the interquartile range with the median, and whiskers follow the standard boxplot convention. Horizontal dashed lines mark the operational AP cutoffs used to define SA/no-SA labels (AP = 1.65 and AP = 1.8).

Modeling Settings

All data, code and artifacts generated from this work are present in https://github.com /tummfm/pepmorph. The used conda environment in development is provided in `environment.yml`.

Raw and processed datasets are in `data/raw` and `data/processed`. Deterministic train/-val/test splits are stored in `data/splits/` (split seed = 42). Training and evaluation scripts also accept a `-seed` argument (default 42) and set deterministic seeds for Python, NumPy, and PyTorch. Stochastic descriptor masking uses `mask_prob` = 0.5 and is also seeded.

Paper hyperparams are summarized in Table 2. In CVAE training, length is normalized by the maximum FASTA length (10), and binary and continuous descriptors use separate reconstruction terms weighted by `lambda_bin` and `lambda_cont`. The full configuration is versioned in `src/pepmorph/modeling/masked_cvae/config.yaml`. Pretrained weights are provided in `artifacts/models/`.

Table 2: **Training hyperparameters.** Hyperparameter–value pairs used to train the AP predictor (used for filtering) and the main PepMorph masked CVAE model (used for sequence generation).

Model	Hyperparameter	Value
AP predictor	batch_size	1024
AP predictor	epochs	5
AP predictor	learning_rate	1e-3
Masked CVAE	latent_dim	24
Masked CVAE	encoder_hidden_dim / decoder_hidden_dim	256 / 256
Masked CVAE	num_encoder_layers / num_decoder_layers	2 / 2
Masked CVAE	nhead	8
Masked CVAE	dropout	0.1
Masked CVAE	batch_size	2048
Masked CVAE	num_epochs	250
Masked CVAE	learning_rate	1e-3
Masked CVAE	weight_decay	1e-4
Masked CVAE	kl_weight (beta)	0.05
Masked CVAE	warmup_epochs	100
Masked CVAE	n_cycles	10
Masked CVAE	mask_prob	0.5
Masked CVAE	random_augmentation	15000 / epoch
Masked CVAE	lambda_bin / lambda_cont	2.0 / 0.5
Masked CVAE	sampler_beta_weight / sampler_sa_weight	10.0 / 2.0

Generation Analysis

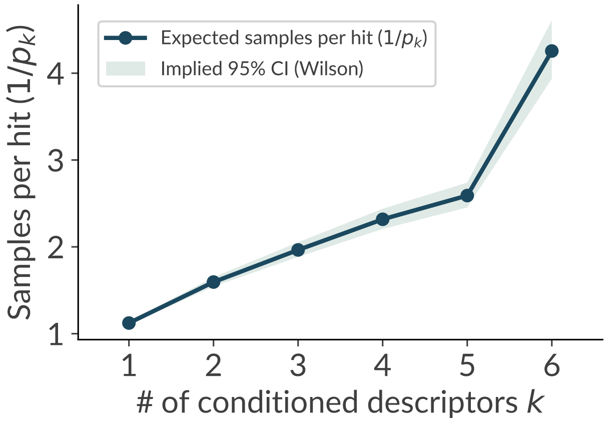

Figure 7: **Sampling cost versus number of conditioned descriptors.** Expected number of sampled peptides required to obtain one fully matching candidate as a function of the number of conditioned descriptors k. For each k, we estimate the empirical success probability p_k as the fraction of generated samples satisfying *all targeted* descriptors (strict conjunction) and report the implied expected samples-per-hit $1/p_k$. The shaded region shows the implied 95% confidence interval obtained by mapping Wilson confidence intervals on p_k to $1/p_k$.

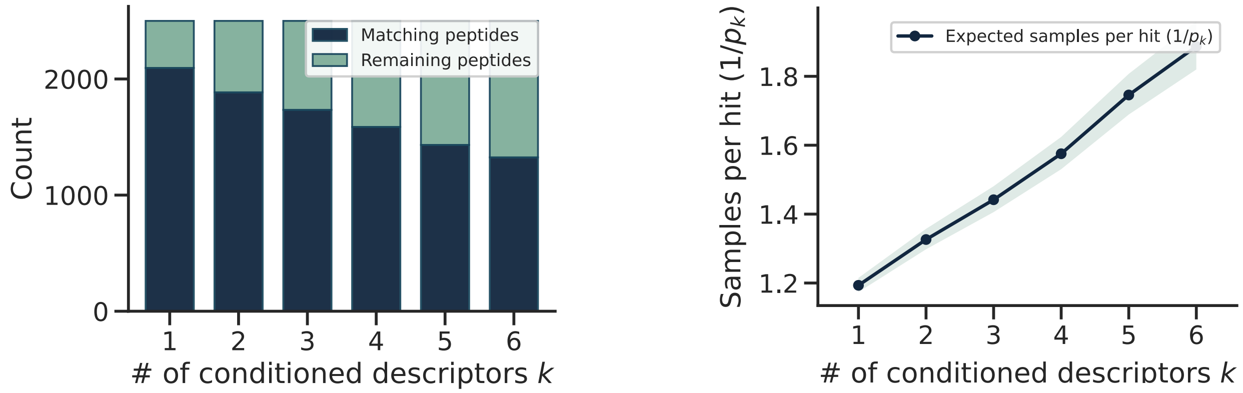

Figure 8: **Held-out test-set conditioning: condition-matching and implied sampling cost.** We instantiate query conditions directly from complete-case held-out test peptides (length 5–10) by selecting random subsets of $k \in \{1, \dots, 6\}$ descriptors and generating one peptide per query under the same decoding settings as in the main evaluation. **Left:** number of generated peptides that match all conditioned targets (dark) versus those that fail at least one target (light) as a function of k. **Right:** implied expected samples per fully matching candidate, $1/p_k$, where p_k is the empirical all-target hit rate at each k; shaded bands denote uncertainty from binomial confidence intervals.

Table 3: **Candidates vs. aggregation-matched descriptor-fail controls (visual outcomes).** Statistical comparison of PepMorph candidates versus aggregation-matched descriptor-fail controls, conditional on aggregation. Wilson confidence intervals are 95%, and Fisher tests are two-sided.

Target	Cohort	Success	Rate (95% CI)	Fisher OR (95% CI), p
Spheres	PepMorph	12/15	0.800 (0.548–0.930)	2.5 (0.46–13.52), 0.410
	Desc.-fail	8/13	0.615 (0.355–0.823)	
Fibrils	PepMorph	13/15	0.867 (0.621–0.963)	7.6 (1.20–48.00), 0.042
	Desc.-fail	6/13	0.462 (0.232–0.709)	
All pooled	PepMorph	25/30	0.833 (0.664–0.927)	4.3 (1.25–14.68), 0.022
	Desc.-fail	14/26	0.538 (0.355–0.712)	

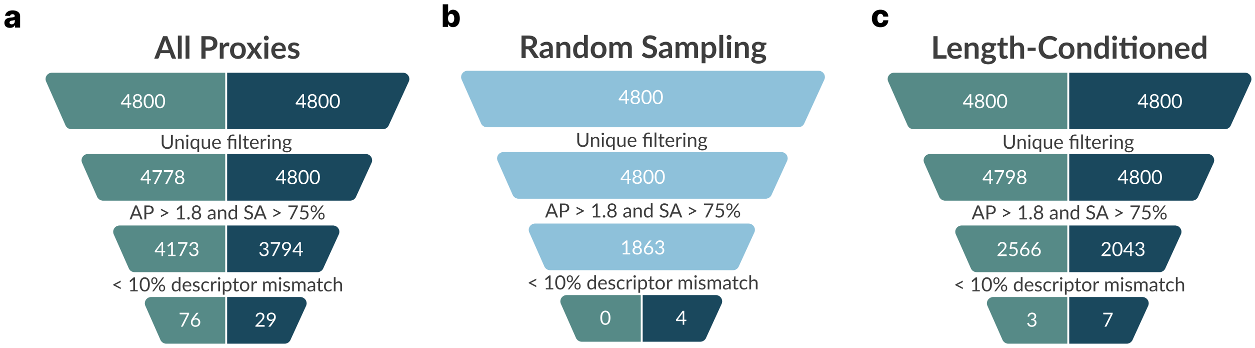

Figure 9: **Filtering funnels for morphology-targeted generation and baseline cohorts.** We compare the post-generation funnel across (a) PepMorph with morphology-proxy conditioning ("All Proxies"), (b) random peptide sampling, and (c) solely length-conditioned generation. Numbers indicate how many unique sequences remain after each stage.

Table 4: **Sample-efficiency under a fixed screening budget.** For each cohort and target regime, we report the number of proxy-compliant hits obtained from $N = 4{,}800$ generated sequences after the full post-generation screening pipeline. Hit rates are binomial proportions with Wilson 95% confidence intervals. Calls-per-hit is reported as 1/HitRate with the interval obtained by inverting the Wilson interval; for zero hits, the point estimate is ∞.

**Cohort**	**Sphere-target**	**Fibril-target**
PepMorph	Hits: 76 / 4,800<br>HitRate: 0.0158 [0.0127, 0.0198]<br>Calls/Hit: 63.2 [50.6, 78.9]	Hits: 29 / 4,800<br>HitRate: 0.0060 [0.0042, 0.0087]<br>Calls/Hit: 165.5 [115.4, 237.5]
Length-cond. unconditional	Hits: 3 / 4,800<br>HitRate: 0.0006 [0.0002, 0.0018]<br>Calls/Hit: 1600.0 [544.6, 4704.2]	Hits: 7 / 4,800<br>HitRate: 0.0015 [0.0007, 0.0030]<br>Calls/Hit: 685.7 [332.5, 1415.2]
Random sampling	Hits: 0 / 4,800<br>HitRate: 0.0000 [0.0000, 0.0008]<br>Calls/Hit: ∞ [1250.5, ∞]	Hits: 4 / 4,800<br>HitRate: 0.0008 [0.0003, 0.0021]<br>Calls/Hit: 1200.0 [467.1, 3085.4]

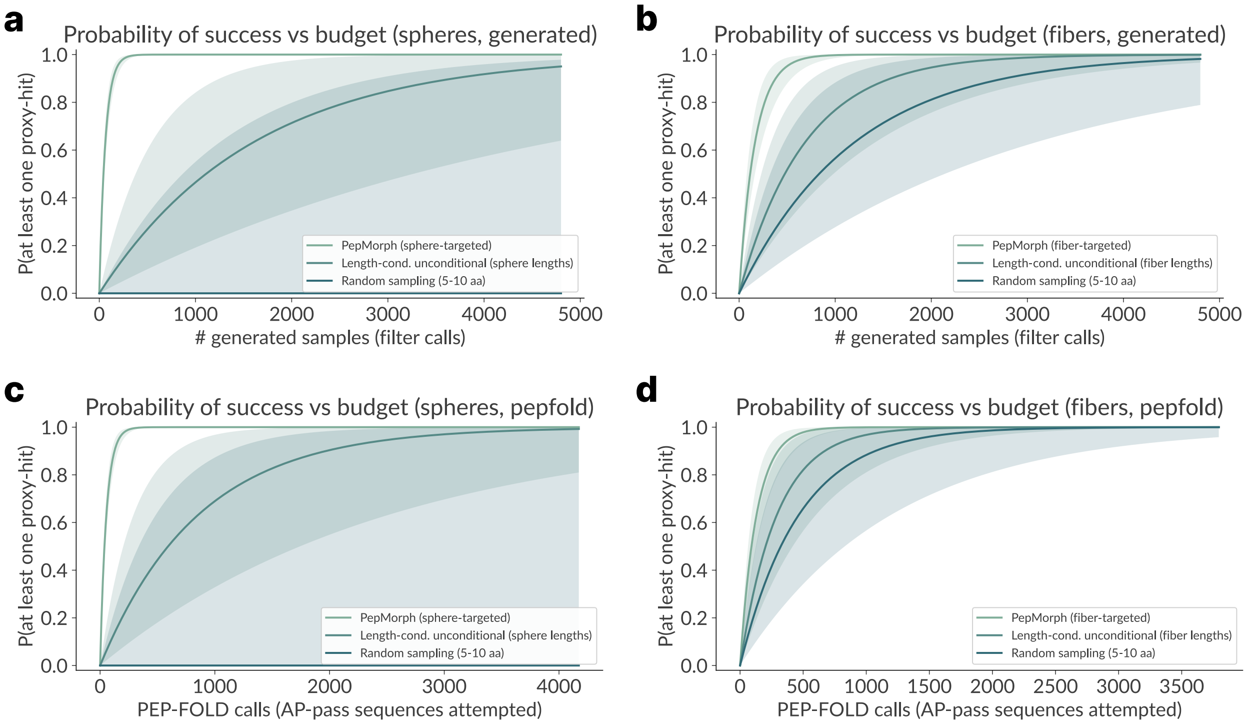

Figure 10: **Budget–success curves for proxy-based discovery (PepMorph vs. baselines).** We report the probability of obtaining at least one proxy-compliant candidate ("proxy-hit") as a function of sampling budget for (a,c) sphere-targeted and (b,d) fibril-targeted discovery. A proxy-hit is defined as a generated sequence that (i) passes aggregation screening (predicted AP $\geq$ 1.8 and SA probability $\geq$ 75%), (ii) yields a valid PEP-FOLD structure for descriptor extraction, and (iii) matches all morphology-proxy windows (within $\pm$10% for continuous proxies; β-strand assignment required for fibrils). Panels (a,b) use # generated samples (filter calls) as budget, while panels (c,d) use # PEP-FOLD calls (AP-pass sequences attempted) as budget. Curves are shown for PepMorph (morphology-proxy conditioning), a length-conditioned unconditional baseline (lengths matched to the target cohorts), and random sampling (uniform 5–10 aa); shaded regions indicate uncertainty propagated from Wilson 95% confidence intervals on the empirical hit rate.

CG-MD Validation

Random Cohort

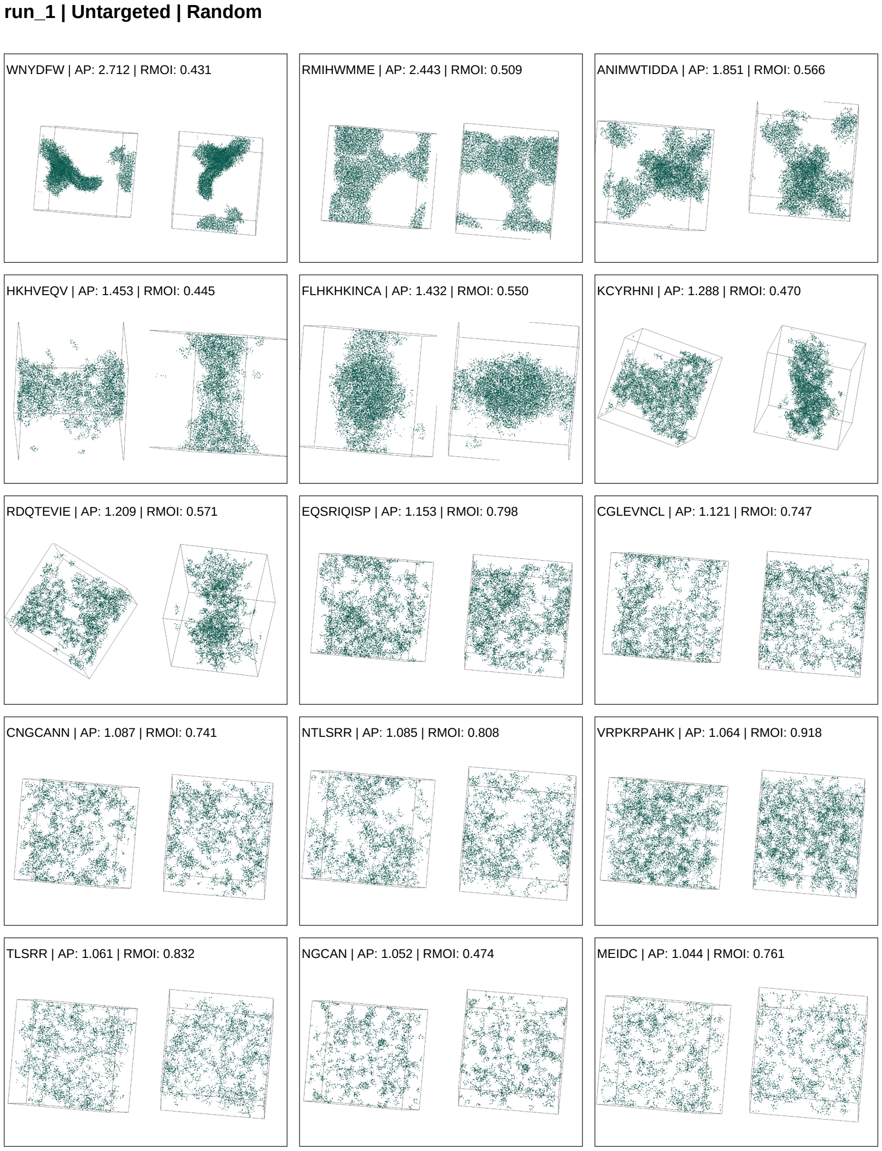

Figure 11: Visual representation, from two different views, of the final frame of the trajectory from the first run for all random targets, with corresponding AP and RMOI reported. Ordered from highest AP to lowest AP peptide in the cohort.

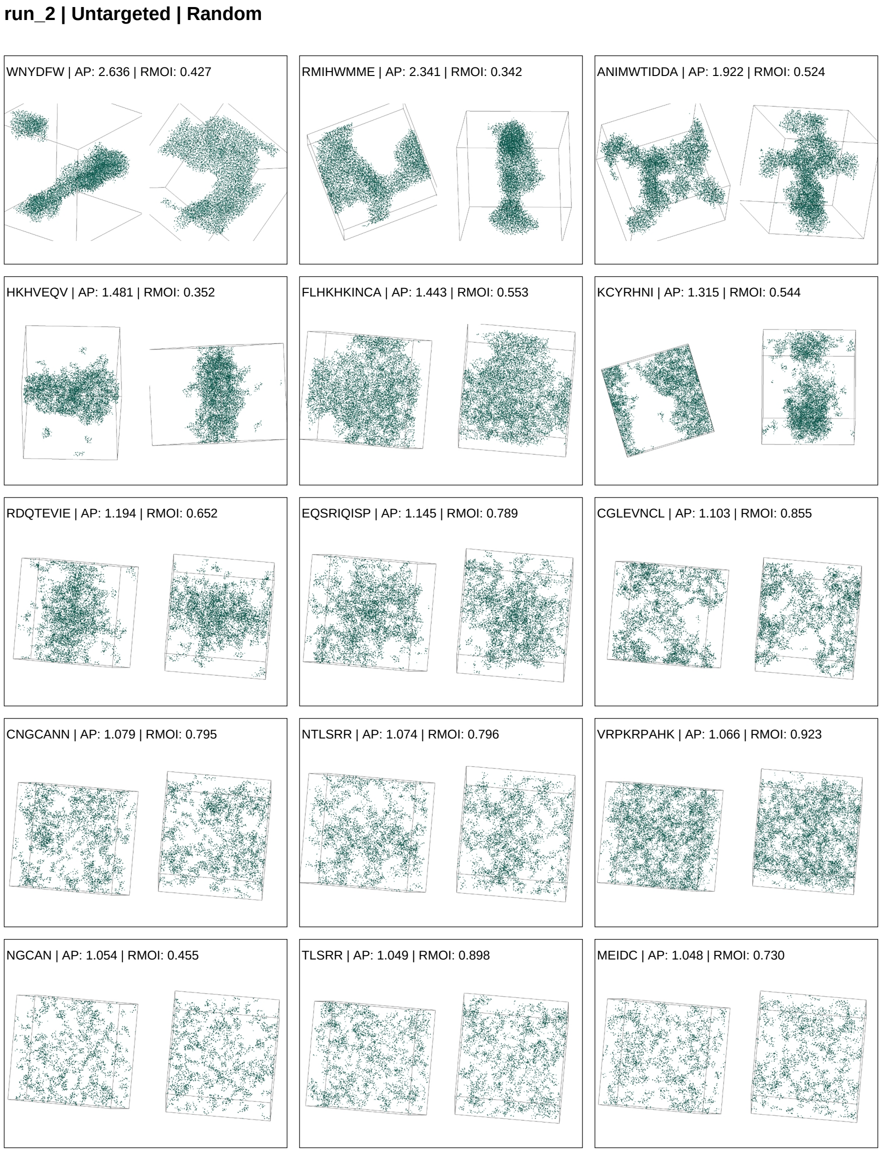

Figure 12: Visual representation, from two different views, of the final frame of the trajectory from the second run for all random targets, with corresponding AP and RMOI reported. Ordered from highest AP to lowest AP peptide in the cohort.

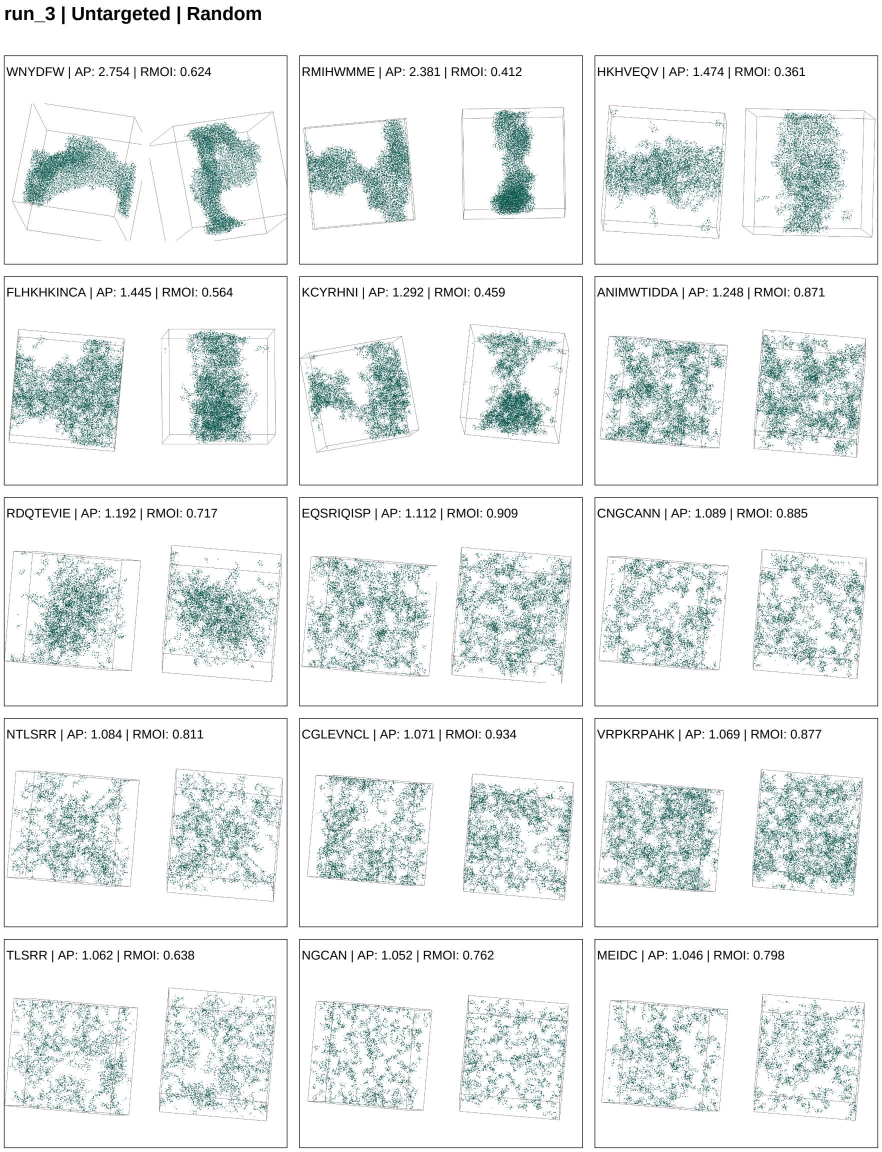

Figure 13: Visual representation, from two different views, of the final frame of the trajectory from the third run for all random targets, with corresponding AP and RMOI reported. Ordered from highest AP to lowest AP peptide in the cohort.

Table 5: Per-peptide results for random peptides. Reported AP, RMOI for all runs, as well as aggregated information. Visual morphology is reported per run when available.

**Peptide**	**Run**	**AP**	**RMOI**	**Mean AP**	**Mean RMOI**	**Morphology by RMOI (run)**	**Morphology by RMOI (agg)**	**Visual Morph Description (run)**	**Morphology Visually (run)**	**Morphology Visually (agg)**
	run$_1$	2.712	0.431			✗		curved aggregate	✗	
WNYDFW	run$_2$	2.636	0.427	2.701	0.494	✗	✗	curved aggregate	✗	✗
	run$_3$	2.754	0.624			✗		curved fiber	fiber	
	run$_1$	2.443	0.509			✗		porous aggregate	✗	
RMIHWMME	run$_2$	2.341	0.342	2.388	0.421	fiber	✗	porous aggregate	✗	✗
	run$_3$	2.381	0.412			✗		intertwined fibers	fiber	
	run$_1$	1.851	0.566			✗		amorphous	✗	
ANIMWTIDDA	run$_2$	1.922	0.524	1.673	0.653	✗	✗	amorphous	✗	✗
	run$_3$	1.248	0.871			✗			✗	
	run$_1$	1.453	0.445			✗			✗	
HKHVEQV	run$_2$	1.481	0.352	1.469	0.386	✗	✗		✗	✗
	run$_3$	1.474	0.361			✗			✗	
	run$_1$	1.432	0.550			✗			✗	
FLHKHKINCA	run$_2$	1.443	0.553	1.440	0.556	✗	✗		✗	✗
	run$_3$	1.445	0.564			✗			✗	
	run$_1$	1.288	0.470			✗			✗	
KCYRHNI	run$_2$	1.315	0.544	1.299	0.491	✗	✗		✗	✗
	run$_3$	1.292	0.459			✗			✗	
	run$_1$	1.209	0.571			✗			✗	
RDQTEVIE	run$_2$	1.194	0.652	1.198	0.647	✗	✗		✗	✗
	run$_3$	1.192	0.717			✗			✗	
	run$_1$	1.153	0.798			✗			✗	
EQSRIQISP	run$_2$	1.145	0.789	1.137	0.832	✗	✗		✗	✗
	run$_3$	1.112	0.909			✗			✗	
	run$_1$	1.121	0.747			✗			✗	
CGLEVNCL	run$_2$	1.103	0.855	1.098	0.845	✗	✗		✗	✗
	run$_3$	1.071	0.934			✗			✗	
	run$_1$	1.087	0.741			✗			✗	
CNGCANN	run$_2$	1.079	0.795	1.085	0.807	✗	✗		✗	✗
	run$_3$	1.089	0.885			✗			✗	
	run$_1$	1.085	0.808			✗			✗	
NTLSRR	run$_2$	1.074	0.796	1.081	0.805	✗	✗		✗	✗
	run$_3$	1.084	0.811			✗			✗	
	run$_1$	1.064	0.918			✗			✗	
VRPKRPAHK	run$_2$	1.066	0.923	1.067	0.906	✗	✗		✗	✗
	run$_3$	1.069	0.877			✗			✗	
	run$_1$	1.061	0.832			✗			✗	
TLSRR	run$_2$	1.049	0.898	1.057	0.789	✗	✗		✗	✗
	run$_3$	1.062	0.638			✗			✗	
	run$_1$	1.052	0.474			✗			✗	
NGCAN	run$_2$	1.054	0.455	1.053	0.564	✗	✗		✗	✗
	run$_3$	1.052	0.762			✗			✗	
	run$_1$	1.044	0.761			✗			✗	
MEIDC	run$_2$	1.048	0.730	1.046	0.763	✗	✗		✗	✗
	run$_3$	1.046	0.798			✗			✗	

Unconditional Cohort

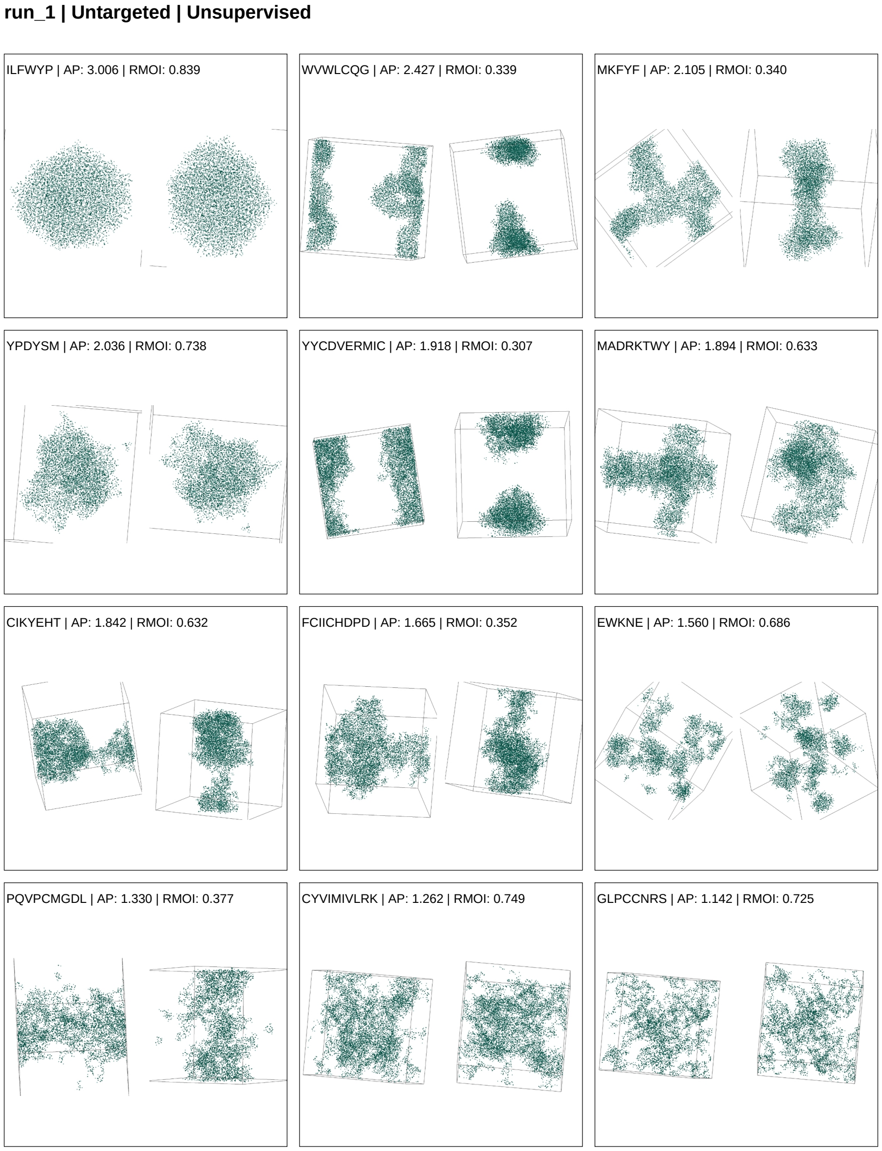

Figure 14: Visual representation, from two different views, of the final frame of the trajectory from the first run for all unconditional targets, with corresponding AP and RMOI reported. Ordered from highest AP to lowest AP peptide in the cohort.

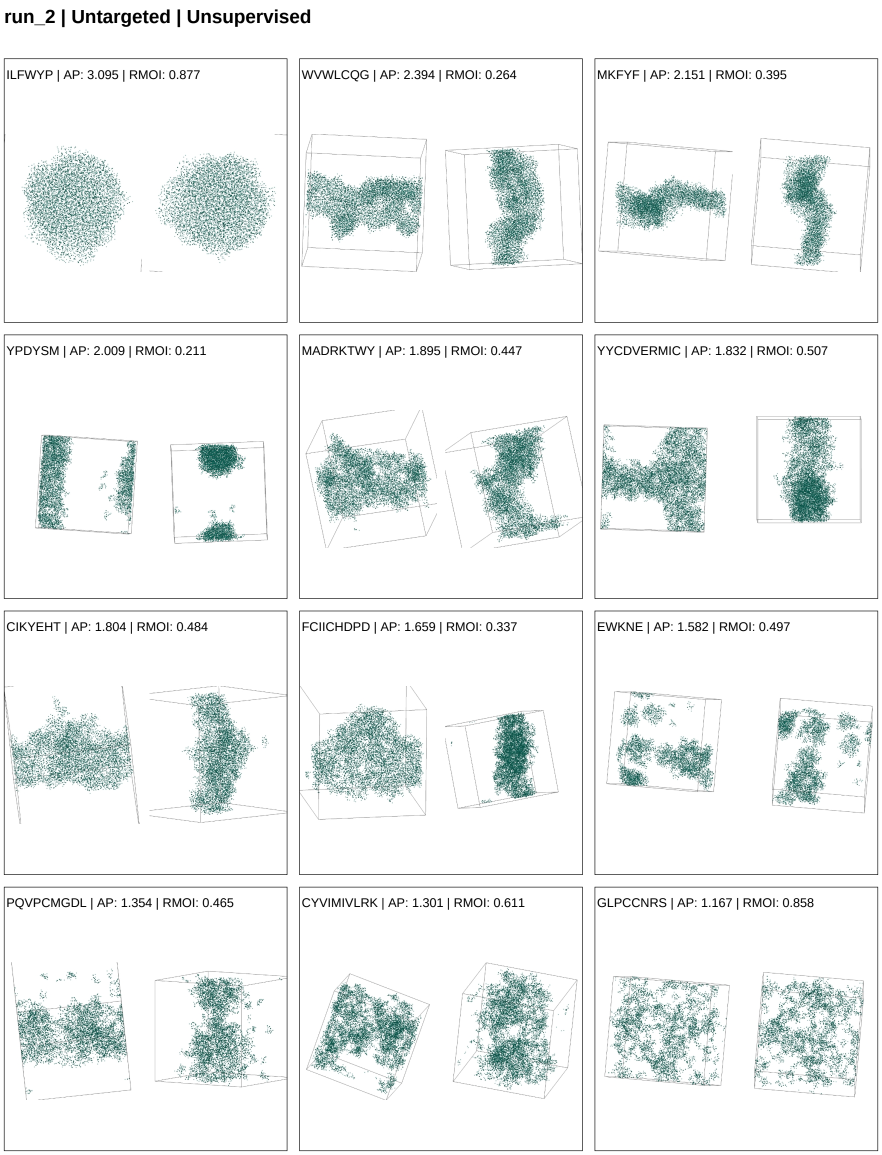

Figure 15: Visual representation, from two different views, of the final frame of the trajectory from the second run for all unconditional targets, with corresponding AP and RMOI reported. Ordered from highest AP to lowest AP peptide in the cohort.

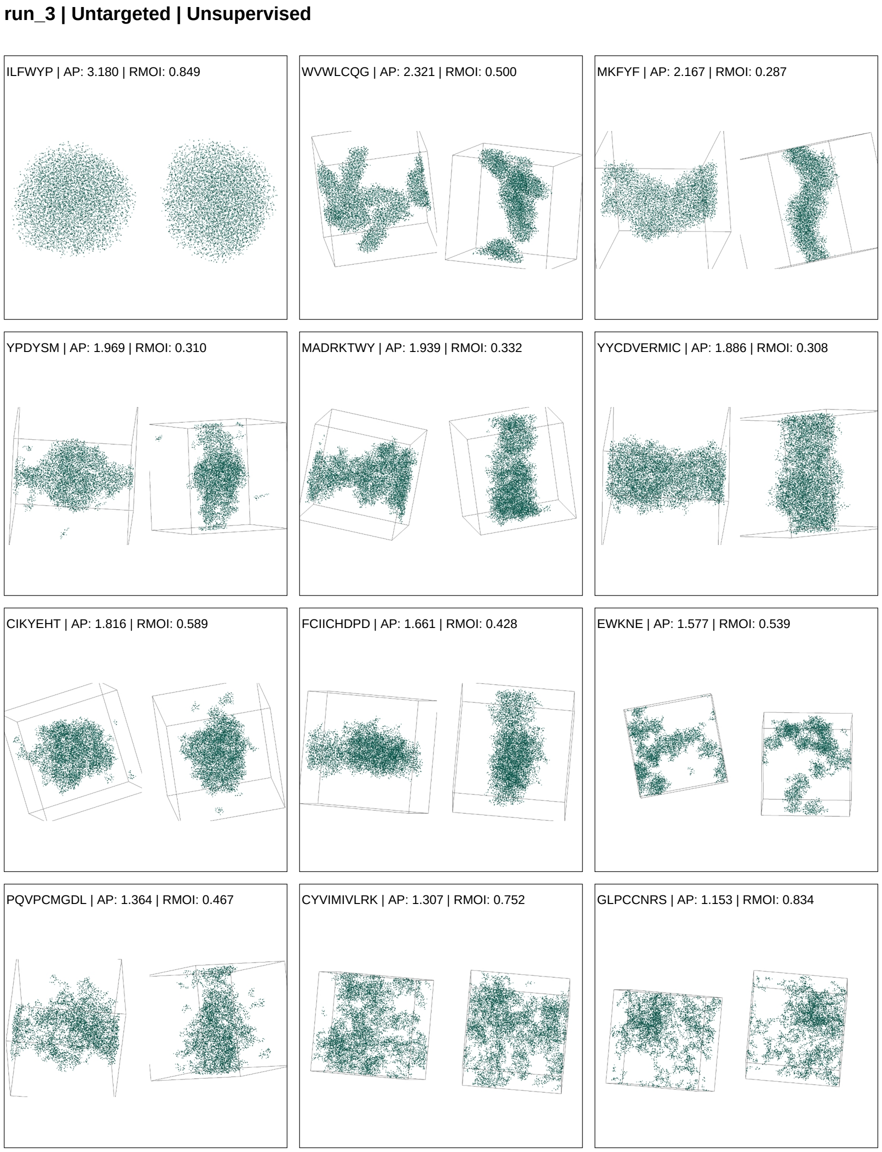

Figure 16: Visual representation, from two different views, of the final frame of the trajectory from the third run for all unconditional targets, with corresponding AP and RMOI reported. Ordered from highest AP to lowest AP peptide in the cohort.

Table 6: Per-peptide results for unconditional peptides. Reported AP, RMOI for all runs, as well as aggregated information. Visual morphology is reported per run when available.

Peptide	Run	AP	RMOI	Mean AP	Mean RMOI	Morphology by RMOI (run)	Morphology by RMOI (agg)	Visual Morph Description (run)	Morphology Visually (run)	Morphology Visually (agg)
	run$_1$	3.006	0.839			sphere		spherical	sphere	
ILFWYP	run$_2$	3.095	0.877	3.094	0.855	sphere	sphere	spherical	sphere	sphere
	run$_3$	3.180	0.849			sphere		spherical	sphere	
	run$_1$	2.427	0.339			fiber		fiber	fiber	
WVWLCQG	run$_2$	2.394	0.264	2.381	0.368	fiber	fiber	fiber	fiber	fiber
	run$_3$	2.321	0.500			✗		branched aggregate	✗	
	run$_1$	2.105	0.340			fiber		fiber	fiber	
MKFYF	run$_2$	2.151	0.395	2.141	0.341	✗	fiber	fiber	fiber	fiber
	run$_3$	2.167	0.287			fiber		fiber	fiber	
YPDYSM	run$_1$	2.036	0.738	2.005	0.420	✗	fiber	irregular spherical aggregate	✗	fiber
	run$_2$	2.009	0.211			fiber		fiber	fiber	
	run$_3$	1.969	0.310			fiber		fiber	fiber	
	run$_1$	1.894	0.633			✗		intertwined fiber	fiber	
MADRKTWY	run$_2$	1.895	0.447	1.909	0.471	✗	✗	curved aggregate	✗	fiber
	run$_3$	1.939	0.332			fiber		irregular fiber	fiber	
	run$_1$	1.918	0.307			fiber		fiber	fiber	
YYCDVERMIC	run$_2$	1.832	0.507	1.879	0.374	✗	fiber	intertwined fiber	fiber	fiber
	run$_3$	1.886	0.308			fiber		fiber	fiber	
	run$_1$	1.842	0.632			✗		disconnected fiber	✗	
CIKYEHT	run$_2$	1.804	0.484	1.821	0.568	✗	✗	fiber	fiber	✗
	run$_3$	1.816	0.589			✗		amorphous	✗	
	run$_1$	1.665	0.352			✗			✗	
FCIICHDPD	run$_2$	1.659	0.337	1.662	0.372	✗	✗		✗	✗
	run$_3$	1.661	0.428			✗			✗	
	run$_1$	1.560	0.686			✗			✗	
EWKNE	run$_2$	1.582	0.497	1.573	0.574	✗	✗		✗	✗
	run$_3$	1.577	0.539			✗			✗	
	run$_1$	1.330	0.377			✗			✗	
PQVPCMGDL	run$_2$	1.354	0.465	1.349	0.436	✗	✗		✗	✗
	run$_3$	1.364	0.467			✗			✗	
	run$_1$	1.262	0.749			✗			✗	
CYVIMIVLRK	run$_2$	1.301	0.611	1.290	0.704	✗	✗		✗	✗
	run$_3$	1.307	0.752			✗			✗	
	run$_1$	1.142	0.725			✗			✗	
GLPCCNRS	run$_2$	1.167	0.858	1.154	0.806	✗	✗		✗	✗
	run$_3$	1.153	0.834			✗			✗	

Spherical-Targeted, Failed AP Screen Cohort

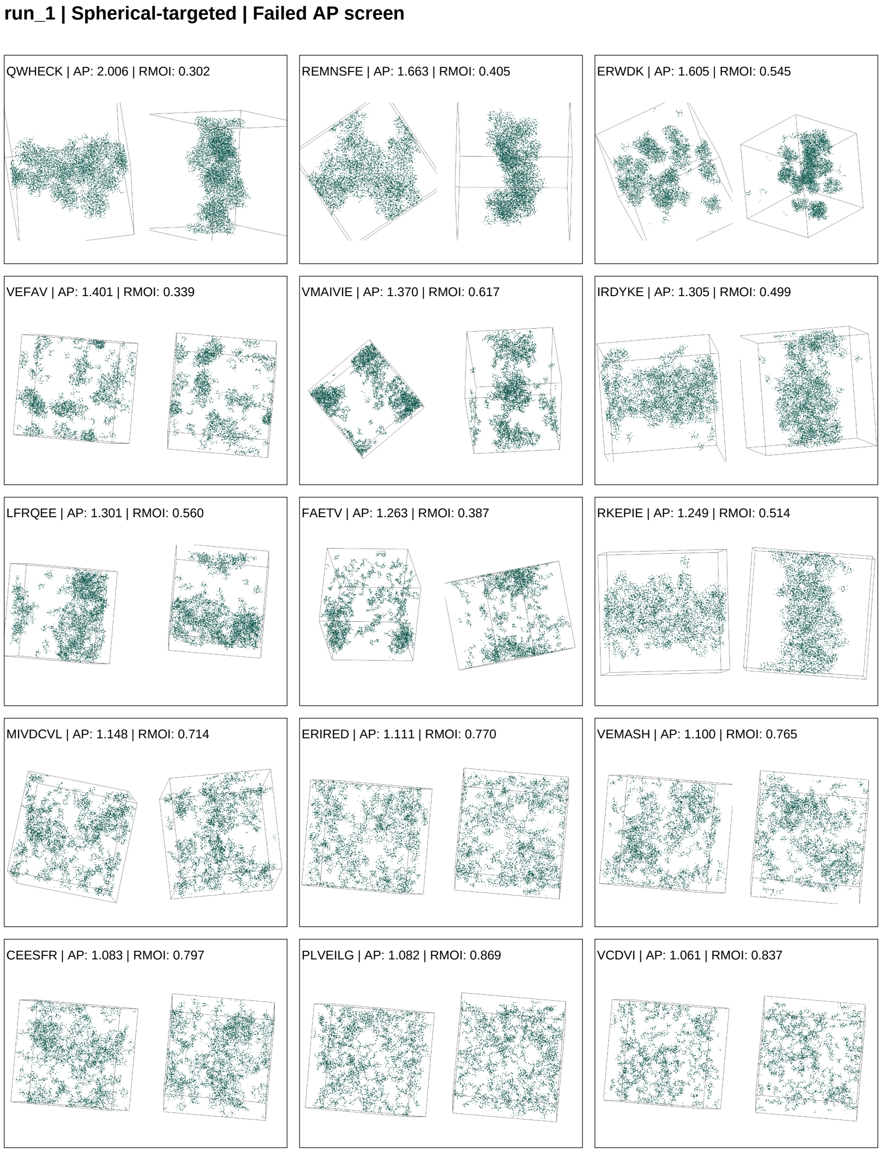

Figure 17: Visual representation, from two different views, of the final frame of the trajectory from the first run for the spherical-targeted cohort failing the AP screen, with AP and RMOI reported. Ordered from highest AP to lowest AP peptide in the cohort.

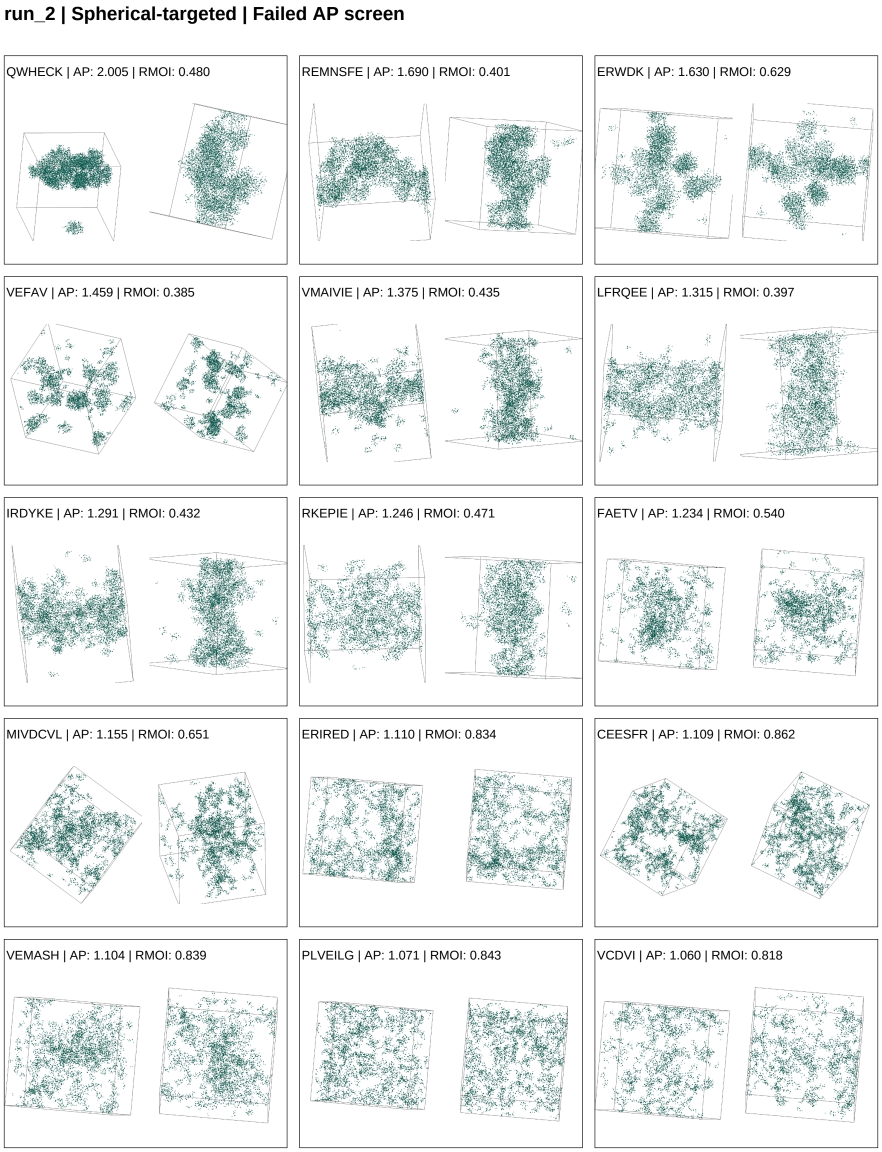

Figure 18: Visual representation, from two different views, of the final frame of the trajectory from the second run for the spherical-targeted cohort failing the AP screen, with AP and RMOI reported. Ordered from highest AP to lowest AP peptide in the cohort.

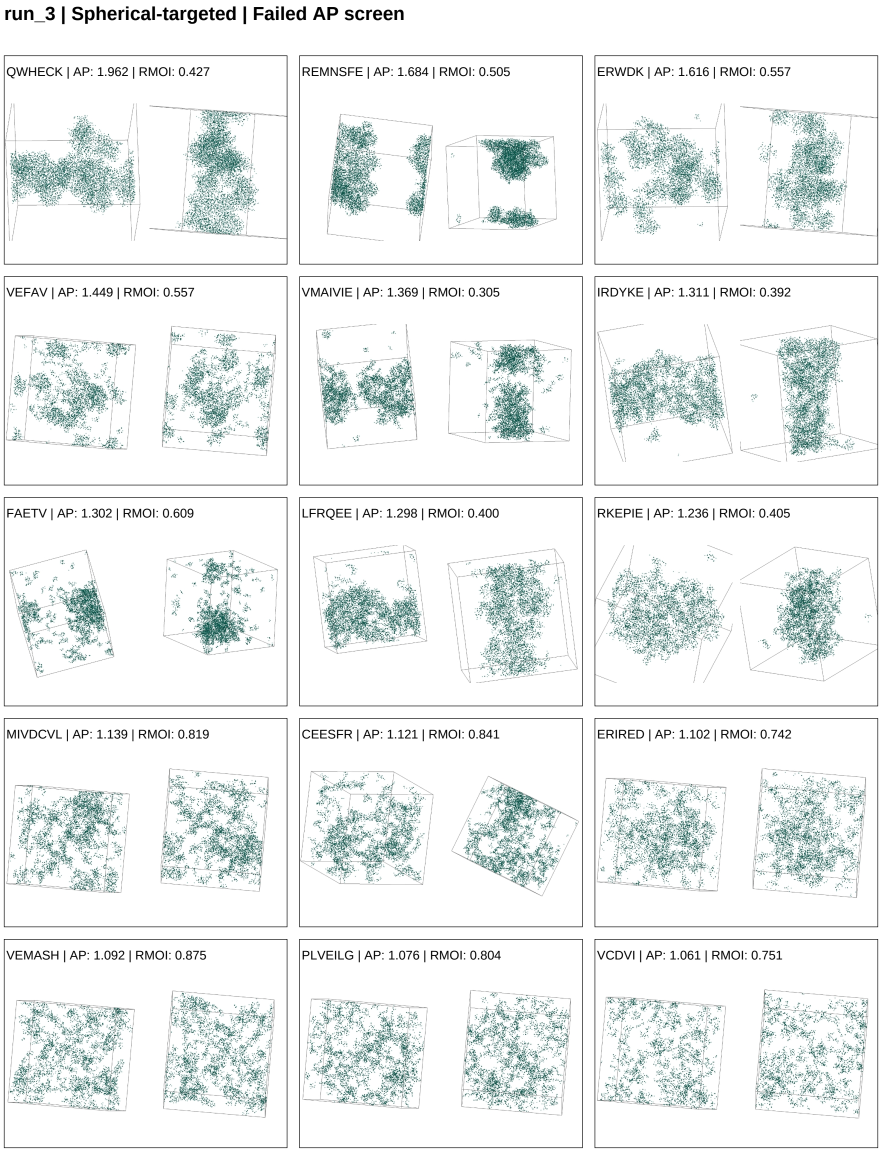

Figure 19: Visual representation, from two different views, of the final frame of the trajectory from the third run for the spherical-targeted cohort failing the AP screen, with AP and RMOI reported. Ordered from highest AP to lowest AP peptide in the cohort.

Table 7: Per-peptide results for sphere targets failing AP screen. Reported AP, RMOI for all runs, as well as aggregated information. Visual morphology is reported per run when available.

**Peptide**	**Run**	**AP**	**RMOI**	**Mean AP**	**Mean RMOI**	**Is Sphere by RMOI (run)**	**Is Sphere by RMOI (agg)**	**Visual Morph Description (run)**	**Is Sphere Visually (run)**	**Is Sphere Visually (agg)**
QWHECK	run_1	2.006	0.302	1.991	0.403	✗	✗		✗	✗
	run_2	2.005	0.480			✗			✗	
	run_3	1.962	0.427			✗			✗	
REMNSFE	run_1	1.663	0.405	1.679	0.437	✗	✗		✗	✗
	run_2	1.690	0.401			✗			✗	
	run_3	1.684	0.505			✗			✗	
ERWDK	run_1	1.605	0.545	1.617	0.577	✗	✗		✗	✗
	run_2	1.630	0.629			✗			✗	
	run_3	1.616	0.557			✗			✗	
VEFAV	run_1	1.401	0.339	1.436	0.427	✗	✗		✗	✗
	run_2	1.459	0.385			✗			✗	
	run_3	1.449	0.557			✗			✗	
VMAIVIE	run_1	1.370	0.617	1.371	0.452	✗	✗		✗	✗
	run_2	1.375	0.435			✗			✗	
	run_3	1.369	0.305			✗			✗	
LFRQEE	run_1	1.301	0.560	1.305	0.453	✗	✗		✗	✗
	run_2	1.315	0.397			✗			✗	
	run_3	1.298	0.400			✗			✗	
IRDYKE	run_1	1.305	0.499	1.302	0.441	✗	✗		✗	✗
	run_2	1.291	0.432			✗			✗	
	run_3	1.311	0.392			✗			✗	
FAETV	run_1	1.263	0.387	1.266	0.512	✗	✗		✗	✗
	run_2	1.234	0.540			✗			✗	
	run_3	1.302	0.609			✗			✗	
RKEPIE	run_1	1.249	0.514	1.244	0.463	✗	✗		✗	✗
	run_2	1.246	0.471			✗			✗	
	run_3	1.236	0.405			✗			✗	
MIVDCVL	run_1	1.148	0.714	1.147	0.728	✗	✗		✗	✗
	run_2	1.155	0.651			✗			✗	
	run_3	1.139	0.819			✗			✗	
ERIRED	run_1	1.111	0.770	1.108	0.782	✗	✗		✗	✗
	run_2	1.110	0.834			✗			✗	
	run_3	1.102	0.742			✗			✗	
CEESFR	run_1	1.083	0.797	1.104	0.833	✗	✗		✗	✗
	run_2	1.109	0.862			✗			✗	
	run_3	1.121	0.841			✗			✗	
VEMASH	run_1	1.100	0.765	1.099	0.826	✗	✗		✗	✗
	run_2	1.104	0.839			✗			✗	
	run_3	1.092	0.875			✗			✗	
PLVEILG	run_1	1.082	0.869	1.076	0.839	✗	✗		✗	✗
	run_2	1.071	0.843			✗			✗	
	run_3	1.076	0.804			✗			✗	
VCDVI	run_1	1.061	0.837	1.061	0.802	✗	✗		✗	✗
	run_2	1.060	0.818			✗			✗	
	run_3	1.061	0.751			✗			✗	

Spherical-Targeted, Failed Descriptor Screen Cohort

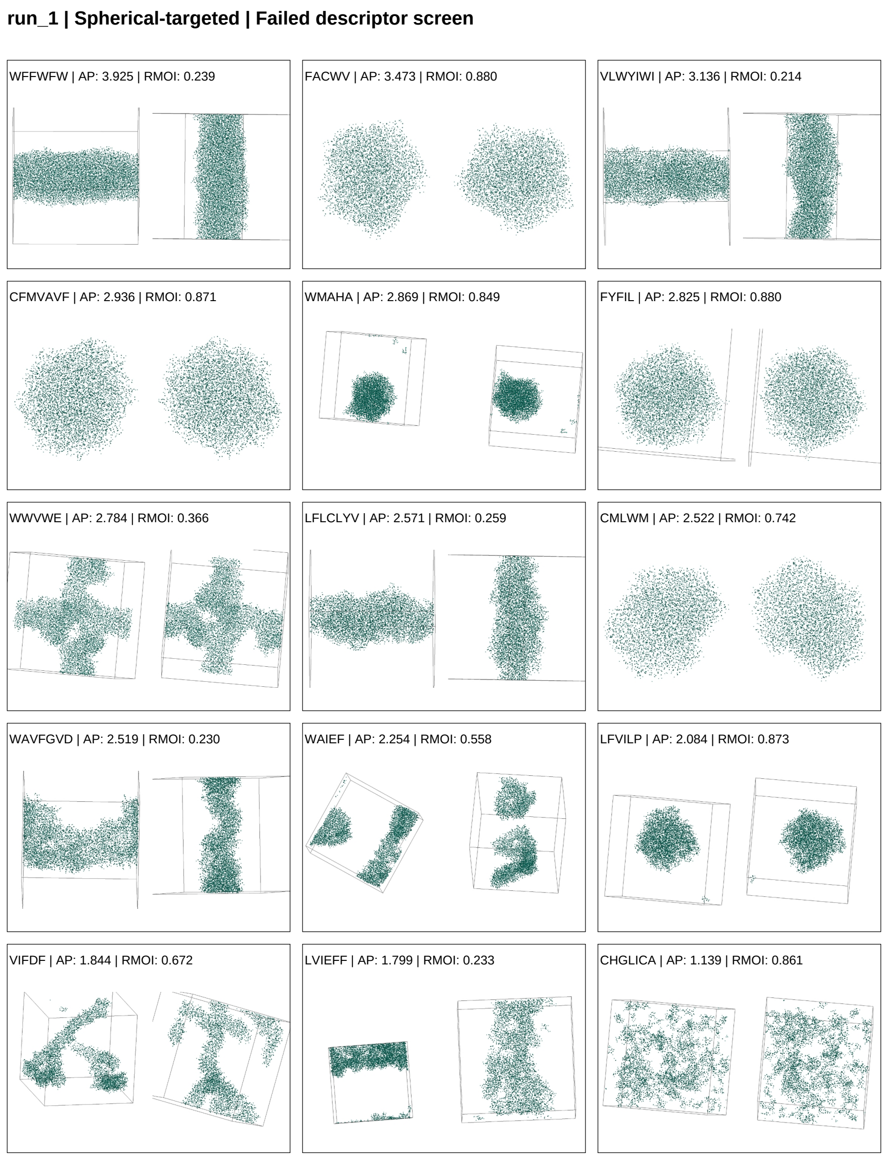

Figure 20: Visual representation, from two different views, of the final frame of the trajectory from the first run for the spherical-targeted cohort failing the descriptor screen, with AP and RMOI reported. Ordered from highest AP to lowest AP peptide in the cohort.

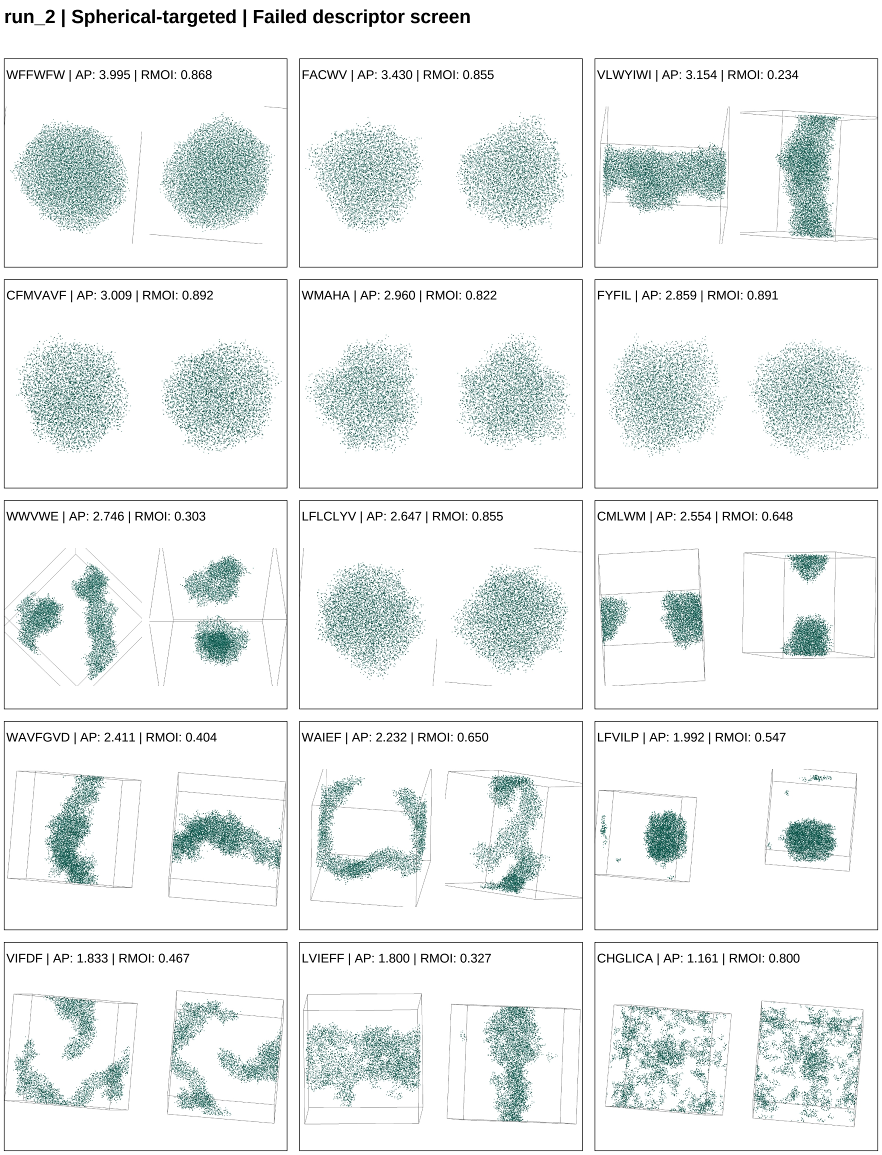

Figure 21: Visual representation, from two different views, of the final frame of the trajectory from the second run for the spherical-targeted cohort failing the descriptor screen, with AP and RMOI reported. Ordered from highest AP to lowest AP peptide in the cohort.

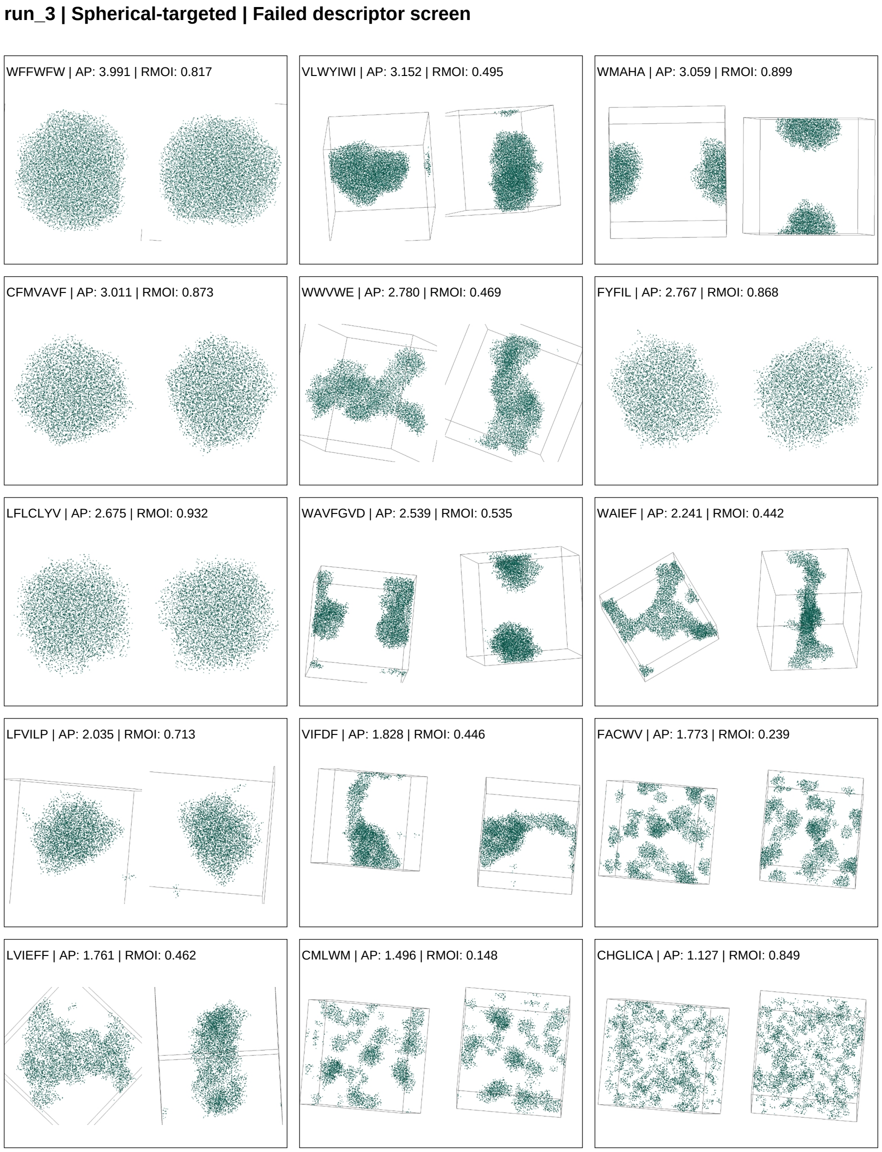

Figure 22: Visual representation, from two different views, of the final frame of the trajectory from the third run for the spherical-targeted cohort failing the descriptor screen, with AP and RMOI reported. Ordered from highest AP to lowest AP peptide in the cohort.

Table 8: Per-peptide results for sphere targets failing descriptor screen. Reported AP, RMOI for all runs, as well as aggregated information. Visual morphology is reported per run when available.

Peptide	Run	AP	RMOI	Mean AP	Mean RMOI	Is Sphere by RMOI (run)	Is Sphere by RMOI (agg)	Visual Morph Description (run)	Is Sphere Visually (run)	Is Sphere Visually (agg)
WFFWFW	run_1	3.925	0.239	3.970	0.641	✗	✓	fiber	✗	✓
	run_2	3.995	0.868			✓		spherical	✓	
	run_3	3.991	0.817			✓		spherical	✓	
VLWYIWI	run_1	3.136	0.214	3.147	0.314	✗	✗	fiber	✗	✗
	run_2	3.154	0.234			✗		fiber	✗	
	run_3	3.152	0.495			✗		elongated aggregate	✗	
CFMVAVF	run_1	2.936	0.871	2.985	0.879	✓	✓	spherical	✓	✓
	run_2	3.009	0.892			✓		spherical	✓	
	run_3	3.011	0.873			✓		spherical	✓	
WMAHA	run_1	2.869	0.849	2.962	0.857	✓	✓	spherical	✓	✓
	run_2	2.960	0.822			✓		spherical	✓	
	run_3	3.059	0.899			✓		spherical	✓	
FACWV	run_1	3.473	0.880	2.892	0.658	✓	✓	spherical	✓	✓
	run_2	3.430	0.855			✓		spherical	✓	
	run_3	1.773	0.239			✗		small aggregates	✗	
FYFIL	run_1	2.825	0.880	2.817	0.880	✓	✓	spherical	✓	✓
	run_2	2.859	0.891			✓		spherical	✓	
	run_3	2.767	0.868			✓		spherical	✓	
WWVWE	run_1	2.784	0.366	2.770	0.379	✗	✗	intertwined fiber	✗	✗
	run_2	2.746	0.303			✗		fiber	✗	
	run_3	2.780	0.469			✗		fiber-like	✗	
LFLCLYV	run_1	2.571	0.259	2.631	0.682	✗	✓	fiber	✗	✓
	run_2	2.647	0.855			✓		spherical	✓	
	run_3	2.675	0.932			✓		spherical	✓	
WAVFGVD	run_1	2.519	0.230	2.490	0.390	✗	✗	fiber	✗	✗
	run_2	2.411	0.404			✗		fiber	✗	
	run_3	2.539	0.535			✗		elongated aggregate	✗	
WAIEF	run_1	2.254	0.558	2.242	0.550	✗	✗	fiber-like	✗	✗
	run_2	2.232	0.650			✗		curved fiber	✗	
	run_3	2.241	0.442			✗		branched aggregate	✗	
CMLWM	run_1	2.522	0.742	2.191	0.513	✗	✗	spherical	✓	✓
	run_2	2.554	0.648			✗		spherical	✓	
	run_3	1.496	0.148			✗		small aggregates	✗	
LFVILP	run_1	2.084	0.873	2.037	0.711	✓	✗	spherical	✓	✓
	run_2	1.992	0.547			✗		spherical	✓	
	run_3	2.035	0.713			✗		spherical	✓	
VIFDF	run_1	1.844	0.672	1.835	0.528	✗	✗	branched aggregate	✗	✗
	run_2	1.833	0.467			✗		branched aggregate	✗	
	run_3	1.828	0.446			✗		curved fiber	✗	
LVIEFF	run_1	1.799	0.233	1.786	0.340	✗	✗		✗	✗
	run_2	1.800	0.327			✗			✗	
	run_3	1.761	0.462			✗			✗	
CHGLICA	run_1	1.139	0.861	1.142	0.836	✗	✗		✗	✗
	run_2	1.161	0.800			✗			✗	
	run_3	1.127	0.849			✗			✗	

Spherical-Targeted, PepMorph Candidates Cohort

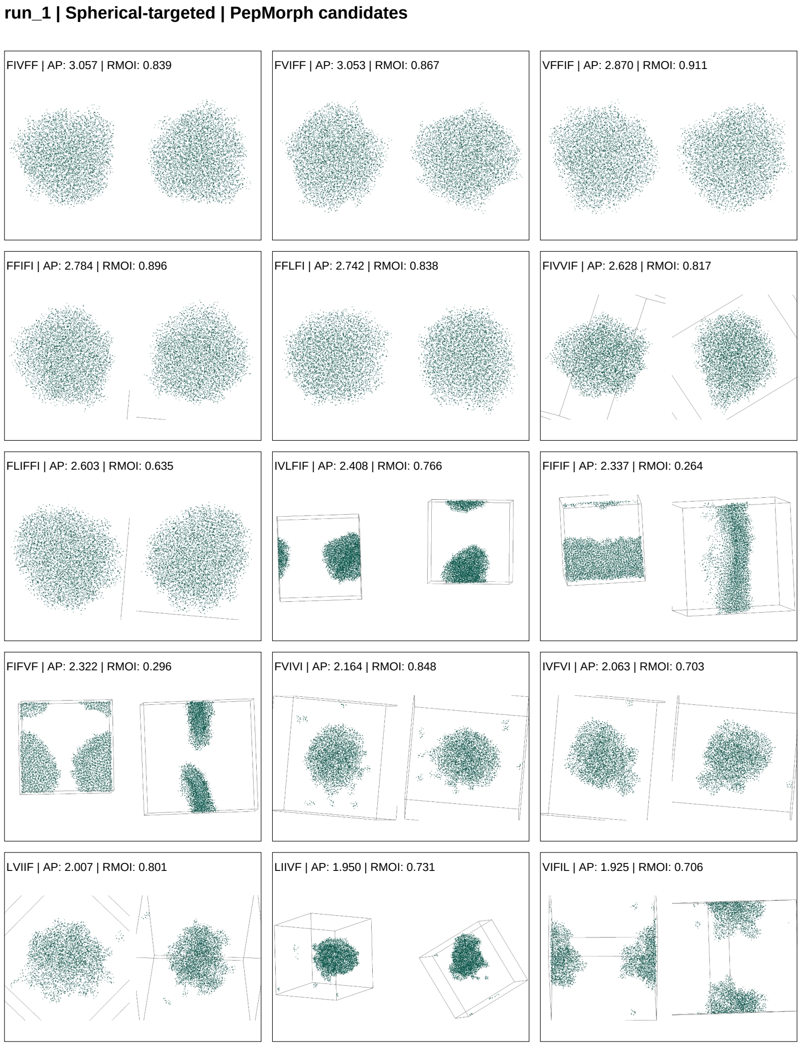

Figure 23: Visual representation, from two different views, of the final frame of the trajectory from the first run for the spherical-targeted PepMorph candidates, with corresponding AP and RMOI reported. Ordered from highest AP to lowest AP peptide in the cohort.

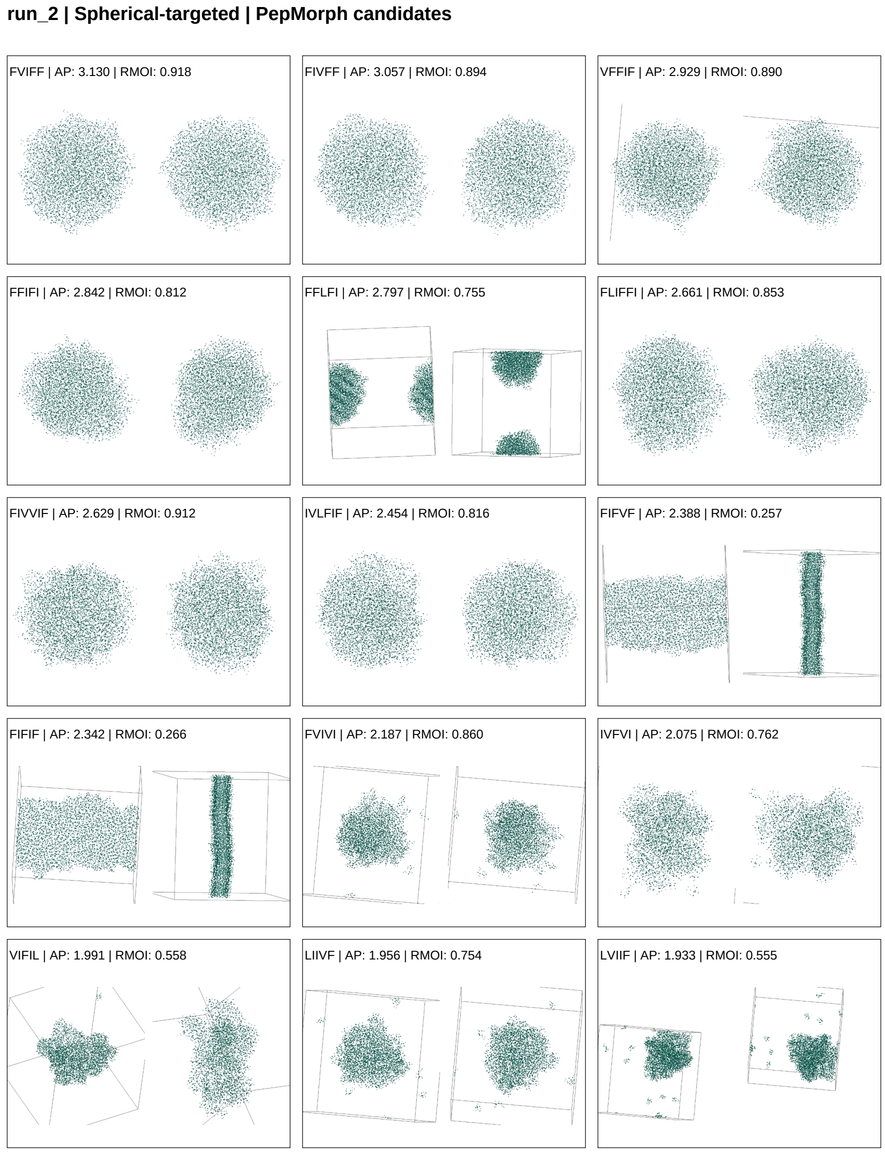

Figure 24: Visual representation, from two different views, of the final frame of the trajectory from the second run for the spherical-targeted PepMorph candidates, with corresponding AP and RMOI reported. Ordered from highest AP to lowest AP peptide in the cohort.

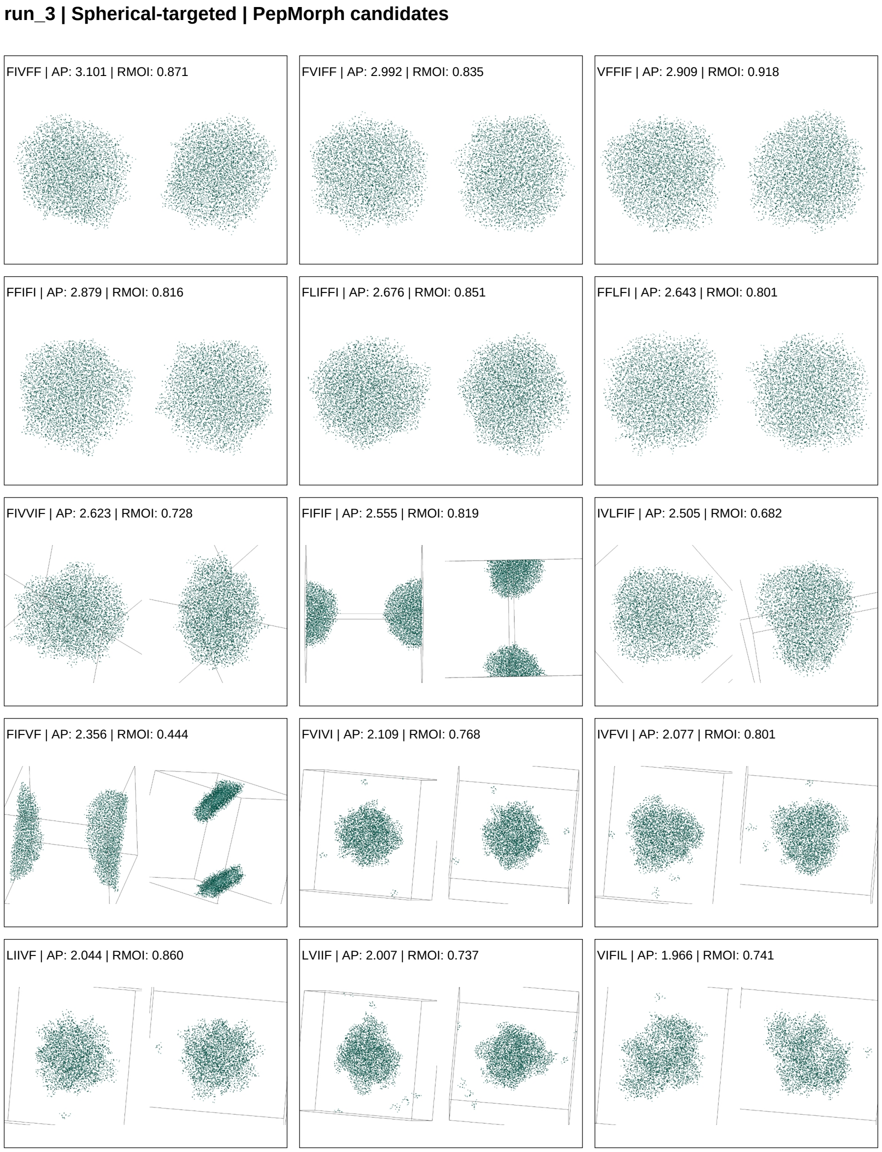

Figure 25: Visual representation, from two different views, of the final frame of the trajectory from the third run for the spherical-targeted PepMorph candidates, with corresponding AP and RMOI reported. Ordered from highest AP to lowest AP peptide in the cohort.

Table 9: Per-peptide results for target spheres. Reported AP, RMOI for all runs, as well as aggregated information. Visual morphology is reported per run when available.

Peptide	Run	AP	RMOI	Mean AP	Mean RMOI	Is Sphere by RMOI (run)	Is Sphere by RMOI (agg)	Visual Morph Description (run)	Is Sphere Visually (run)	Is Sphere Visually (agg)
VFFIF	run_1	2.870	0.911	2.903	0.907	✓	✓	Sphere	✓	✓
	run_2	2.929	0.890			✓		Sphere	✓	
	run_3	2.909	0.918			✓		Sphere	✓	
FVIFF	run_1	3.053	0.867	3.058	0.873	✓	✓	Sphere	✓	✓
	run_2	3.130	0.918			✓		Sphere	✓	
	run_3	2.992	0.835			✓		Sphere	✓	
FIVFF	run_1	3.057	0.839	3.072	0.868	✓	✓	Sphere	✓	✓
	run_2	3.057	0.894			✓		Sphere	✓	
	run_3	3.101	0.871			✓		Sphere	✓	
FFIFI	run_1	2.784	0.896	2.835	0.841	✓	✓	Sphere	✓	✓
	run_2	2.842	0.812			✓		Sphere	✓	
	run_3	2.879	0.816			✓		Sphere	✓	
FVIVI	run_1	2.164	0.848	2.153	0.825	✓	✓	Sphere	✓	✓
	run_2	2.187	0.860			✓		Sphere	✓	
	run_3	2.109	0.768			✓		Sphere	✓	
FIVVIF	run_1	2.628	0.817	2.626	0.819	✓	✓	Sphere	✓	✓
	run_2	2.629	0.912			✓		Sphere	✓	
	run_3	2.623	0.728			✗		Sphere	✓	
FFLFI	run_1	2.742	0.838	2.727	0.798	✓	✓	Sphere	✓	✓
	run_2	2.797	0.755			✓		Sphere	✓	
	run_3	2.643	0.801			✓		Sphere	✓	
LIIVF	run_1	1.950	0.731	1.983	0.781	✗	✓	Sphere	✓	✓
	run_2	1.956	0.754			✓		Sphere	✓	
	run_3	2.044	0.860			✓		Sphere	✓	
FLIFFI	run_1	2.603	0.635	2.647	0.780	✗	✓	Sphere	✓	✓
	run_2	2.661	0.853			✓		Sphere	✓	
	run_3	2.676	0.851			✓		Sphere	✓	
IVFVI	run_1	2.063	0.703	2.072	0.755	✗	✓	Roughly Spherical	✗	✓
	run_2	2.075	0.762			✓		Sphere	✓	
	run_3	2.077	0.801			✓		Sphere	✓	
IVLFIF	run_1	2.408	0.766	2.456	0.754	✓	✓	Sphere	✓	✓
	run_2	2.454	0.816			✓		Sphere	✓	
	run_3	2.505	0.682			✗		Roughly Spherical/Vesicle	✗	
LVIIF	run_1	2.007	0.801	1.982	0.698	✓	✗	Sphere	✓	✓
	run_2	1.933	0.555			✗		Irregular Shape	✗	
	run_3	2.007	0.737			✗		Sphere	✓	
VIFIL	run_1	1.925	0.706	1.961	0.668	✗	✗	Irregular Shape	✗	✗
	run_2	1.991	0.558			✗		Irregular Shape, Elongated	✗	
	run_3	1.966	0.741			✗		Sphere	✓	
FIFIF	run_1	2.337	0.264	2.411	0.450	✗	✗	Sheet	✗	✗
	run_2	2.342	0.266			✗		Sheet	✗	
	run_3	2.555	0.819			✓		Sphere	✓	
FIFVF	run_1	2.322	0.296	2.355	0.332	✗	✗	Sheet	✗	✗
	run_2	2.388	0.257			✗		Sheet	✗	
	run_3	2.356	0.444			✗		Circular Sheet/Disk	✗	

Fibril-Targeted, Failed AP Screen Cohort

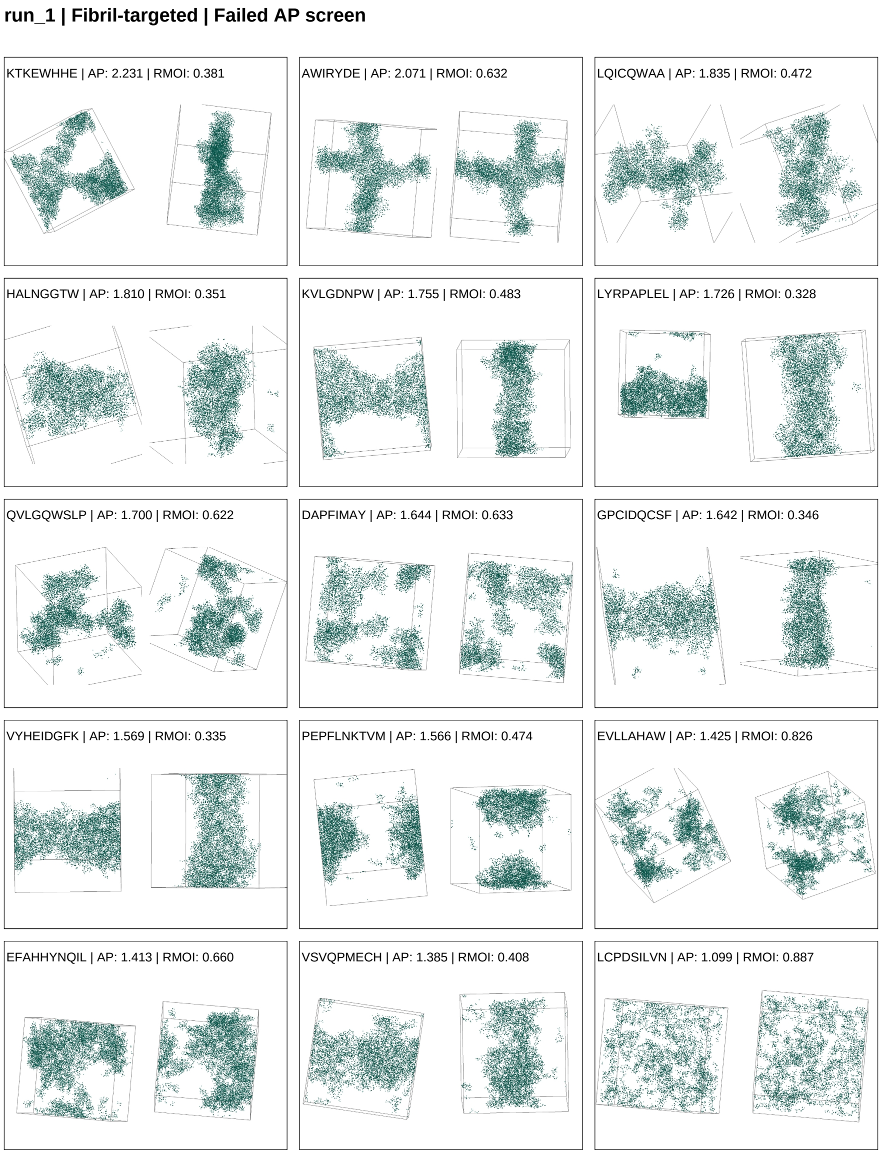

Figure 26: Visual representation, from two different views, of the final frame of the trajectory from the first run for the fibril-targeted cohort failing the AP screen, with AP and RMOI reported. Ordered from highest AP to lowest AP peptide in the cohort.

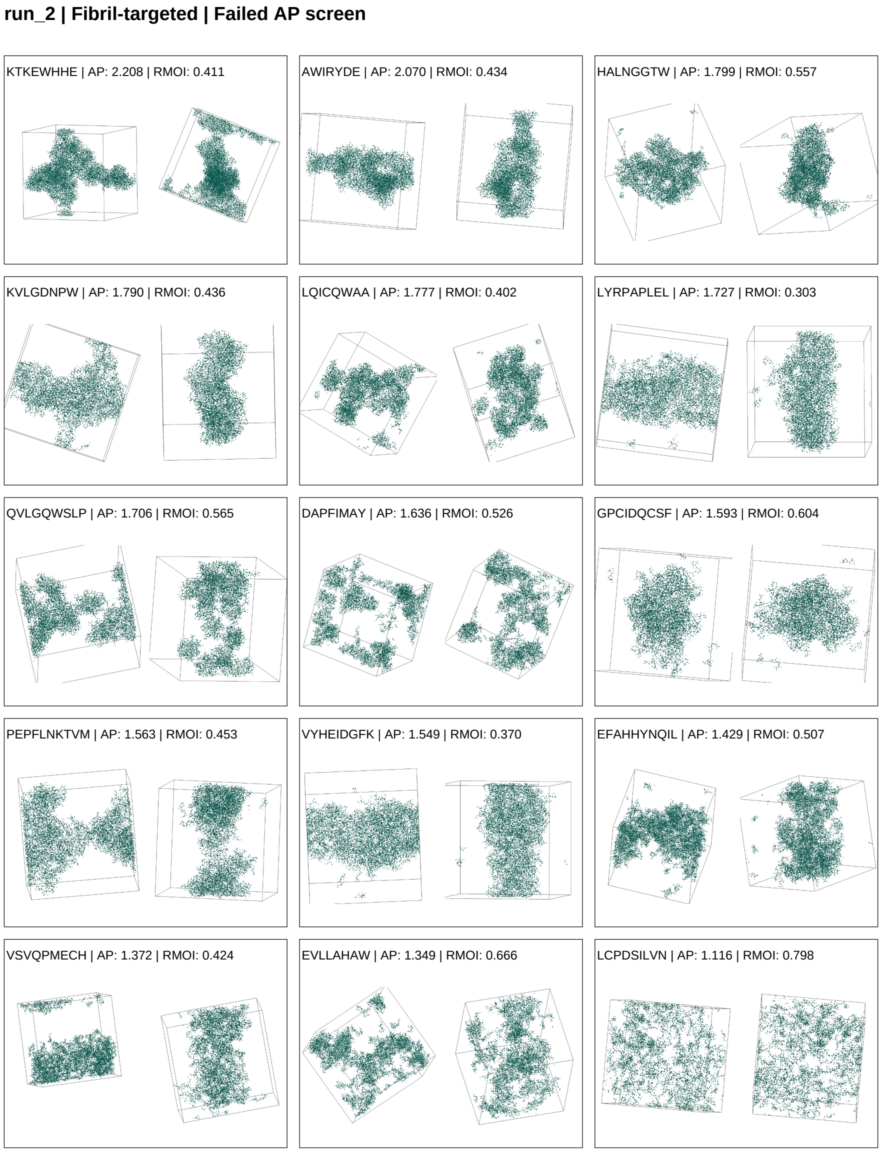

Figure 27: Visual representation, from two different views, of the final frame of the trajectory from the second run for the fibril-targeted cohort failing the AP screen, with AP and RMOI reported. Ordered from highest AP to lowest AP peptide in the cohort.

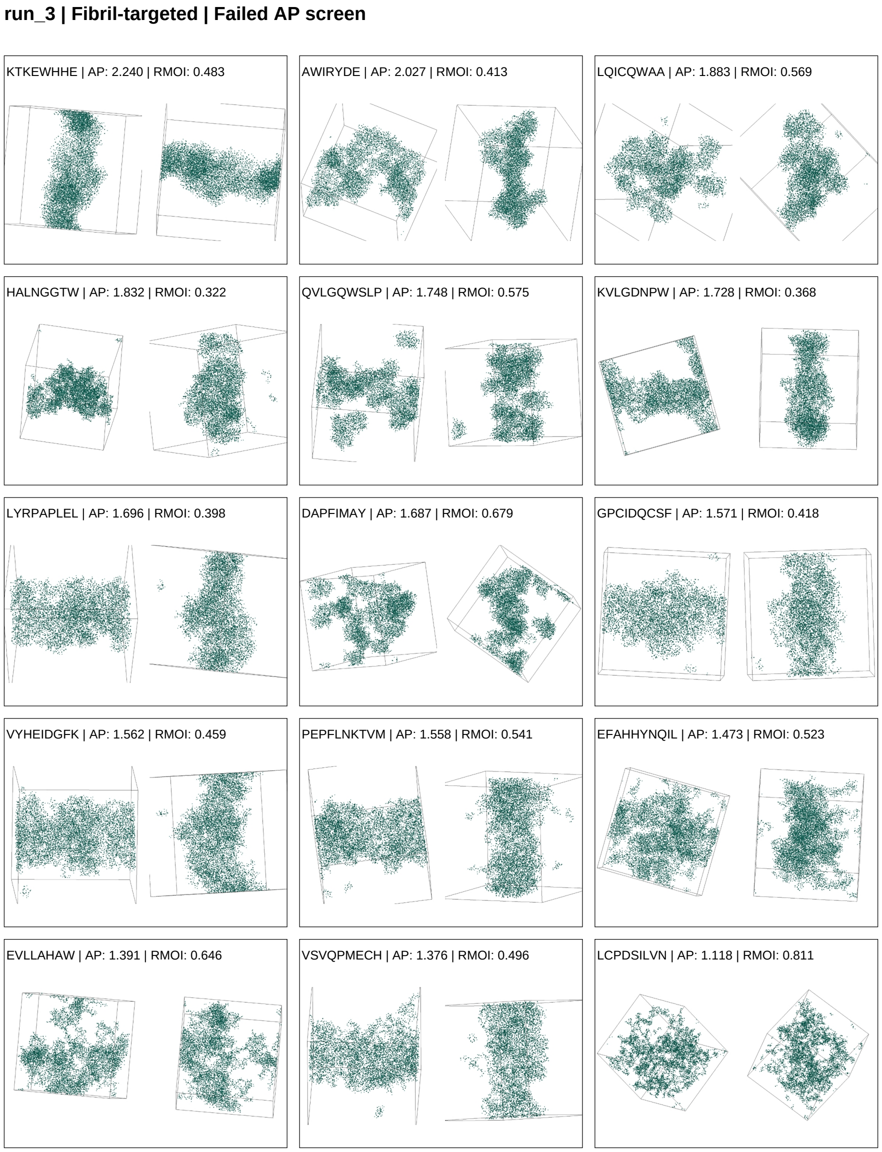

Figure 28: Visual representation, from two different views, of the final frame of the trajectory from the third run for the fibril-targeted cohort failing the AP screen, with AP and RMOI reported. Ordered from highest AP to lowest AP peptide in the cohort.

Table 10: Per-peptide results for target fibers failing AP screen. Reported AP, RMOI for all runs, as well as aggregated information. Visual morphology is reported per run when available.

**Peptide**	**Run**	**AP**	**RMOI**	**Mean AP**	**Mean RMOI**	**Is Fiber by RMOI (run)**	**Is Fiber by RMOI (agg)**	**Visual Morph Description (run)**	**Is Fiber Visually (run)**	**Is Fiber Visually (agg)**
	run$_1$	2.231	0.381			✗		intertwined fibers	✓	
KTKEWHHE	run$_2$	2.208	0.411	2.226	0.425	✗	✗	disconnected fibers	✗	✓
	run$_3$	2.240	0.483			✗		fiber	✓	
	run$_1$	2.071	0.632			✗		intertwined fibers	✓	
AWIRYDE	run$_2$	2.070	0.434	2.056	0.493	✗	✗	fiber	✓	✓
	run$_3$	2.027	0.413			✗		curved fiber	✓	
	run$_1$	1.835	0.472			✗		amorphous	✗	
LQICQWAA	run$_2$	1.777	0.402	1.832	0.481	✗	✗	amorphous	✗	✗
	run$_3$	1.883	0.569			✗		amorphous	✗	
HALNGGTW	run$_1$	1.810	0.351	1.814	0.410	✗	✗	very irregular extended aggregate	✗	✗
	run$_2$	1.799	0.557			✗		elongated aggregate	✗	
	run$_3$	1.832	0.322			✓		curved fiber	✓	
	run$_1$	1.755	0.483			✗			✗	
KVLGDNPW	run$_2$	1.790	0.436	1.757	0.429	✗	✗		✗	✗
	run$_3$	1.728	0.368			✗			✗	
	run$_1$	1.700	0.622			✗			✗	
QVLGQWSLP	run$_2$	1.706	0.565	1.718	0.587	✗	✗		✗	✗
	run$_3$	1.748	0.575			✗			✗	
	run$_1$	1.726	0.328			✗		fiber	✓	
LYRPAPLEL	run$_2$	1.727	0.303	1.716	0.343	✗	✗	fiber	✓	✗
	run$_3$	1.696	0.398			✗		fiber	✓	
	run$_1$	1.644	0.633			✗			✗	
DAPFIMAY	run$_2$	1.636	0.526	1.656	0.613	✗	✗		✗	✗
	run$_3$	1.687	0.679			✗			✗	
	run$_1$	1.642	0.346			✗			✗	
GPCIDQCSF	run$_2$	1.593	0.604	1.602	0.456	✗	✗		✗	✗
	run$_3$	1.571	0.418			✗			✗	
	run$_1$	1.566	0.474			✗			✗	
PEPFLNKTVM	run$_2$	1.563	0.453	1.562	0.489	✗	✗		✗	✗
	run$_3$	1.558	0.541			✗			✗	
	run$_1$	1.569	0.335			✗			✗	
VYHEIDGFK	run$_2$	1.549	0.370	1.560	0.388	✗	✗		✗	✗
	run$_3$	1.562	0.459			✗			✗	
	run$_1$	1.413	0.660			✗			✗	
EFAHHYNQIL	run$_2$	1.429	0.507	1.438	0.563	✗	✗		✗	✗
	run$_3$	1.473	0.523			✗			✗	
	run$_1$	1.425	0.826			✗			✗	
EVLLAHAW	run$_2$	1.349	0.666	1.388	0.712	✗	✗		✗	✗
	run$_3$	1.391	0.646			✗			✗	
	run$_1$	1.385	0.408			✗			✗	
VSVQPMECH	run$_2$	1.372	0.424	1.378	0.443	✗	✗		✗	✗
	run$_3$	1.376	0.496			✗			✗	
	run$_1$	1.099	0.887			✗			✗	
LCPDSILVN	run$_2$	1.116	0.798	1.111	0.832	✗	✗		✗	✗
	run$_3$	1.118	0.811			✗			✗	

Fibril-Targeted, Failed Descriptor Screen Cohort

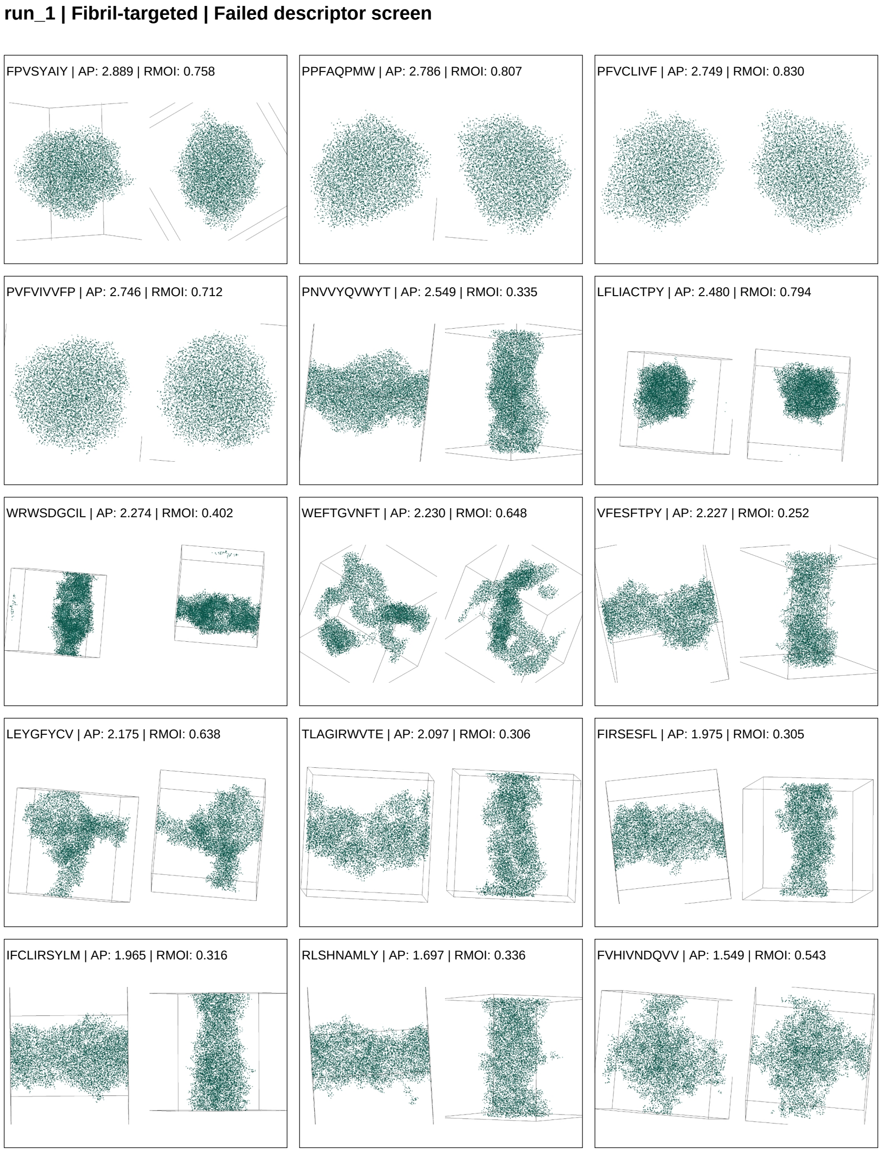

Figure 29: Visual representation, from two different views, of the final frame of the trajectory from the first run for the fibril-targeted cohort failing the descriptor screen, with AP and RMOI reported. Ordered from highest AP to lowest AP peptide in the cohort.

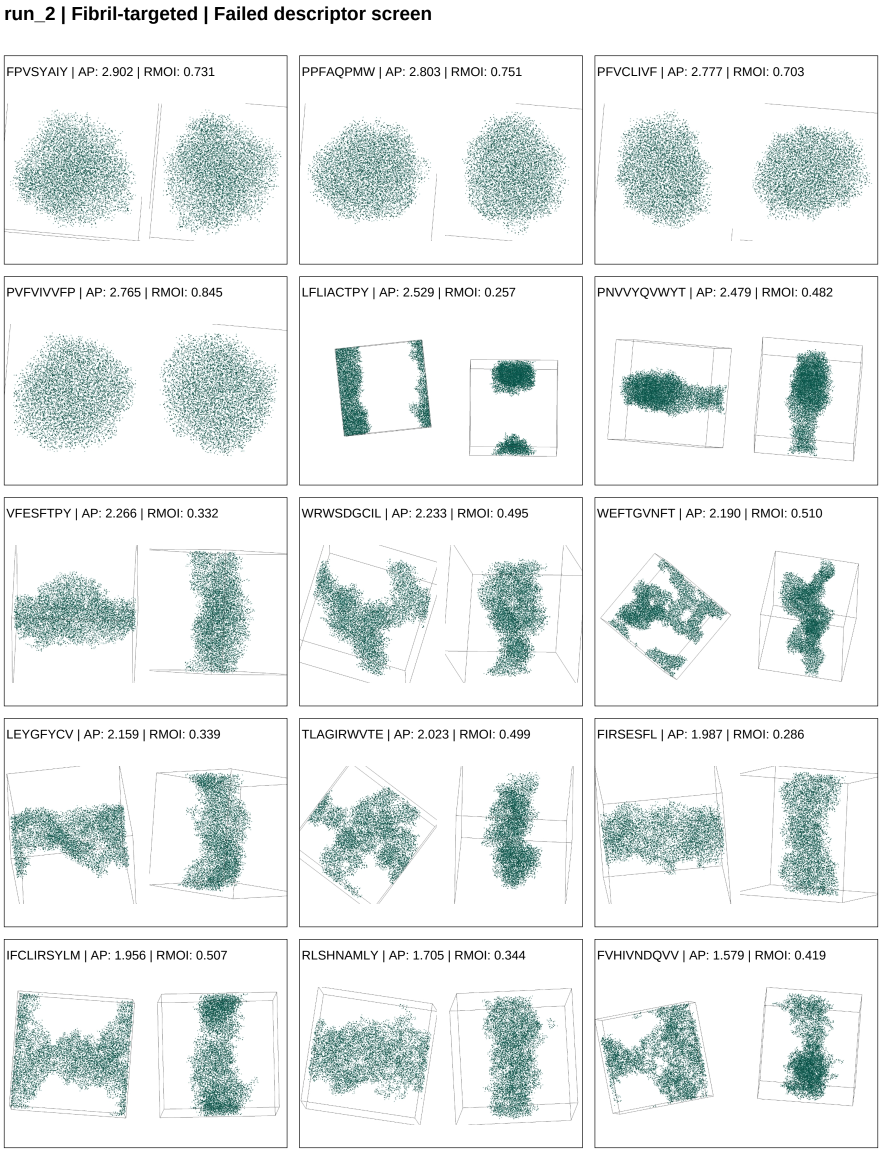

Figure 30: Visual representation, from two different views, of the final frame of the trajectory from the second run for the fibril-targeted cohort failing the descriptor screen, with AP and RMOI reported. Ordered from highest AP to lowest AP peptide in the cohort.

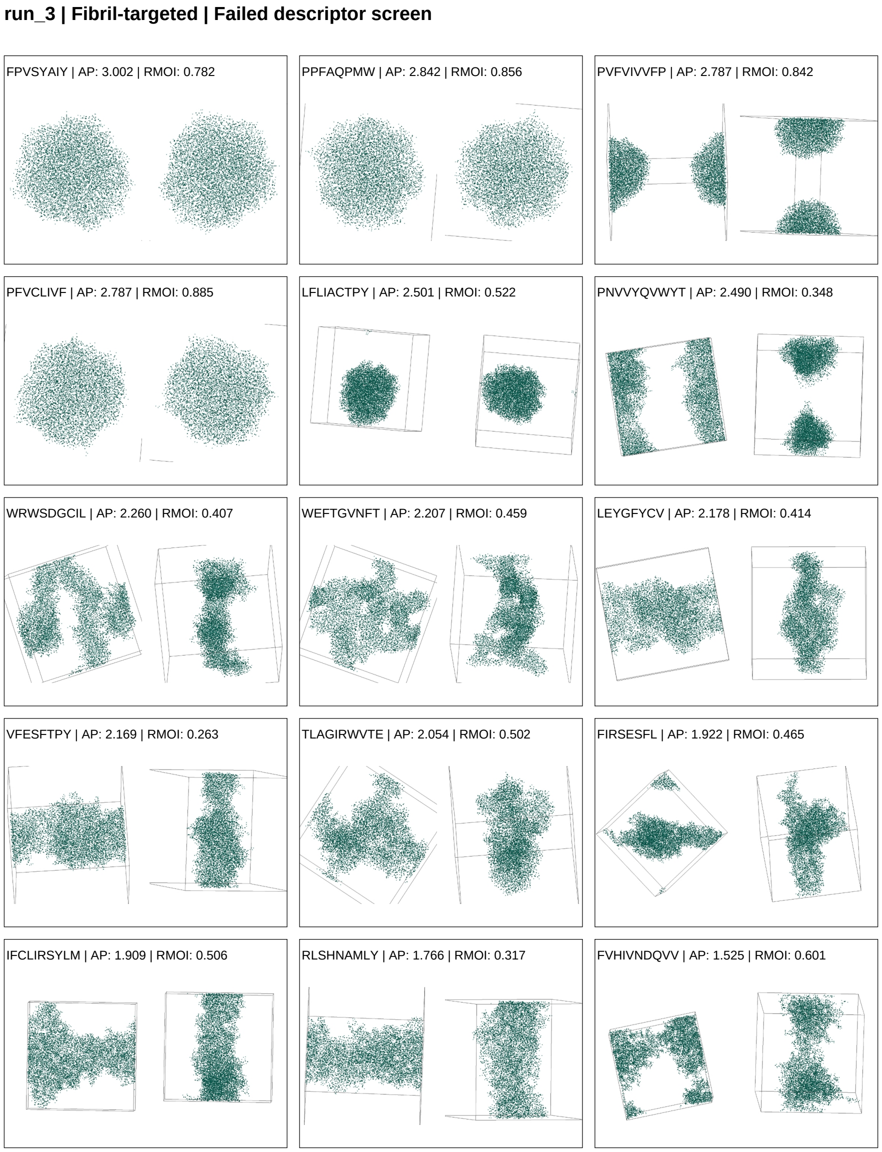

Figure 31: Visual representation, from two different views, of the final frame of the trajectory from the third run for the fibril-targeted cohort failing the descriptor screen, with AP and RMOI reported. Ordered from highest AP to lowest AP peptide in the cohort.

Table 11: Per-peptide results for target fibers failing descriptor screen. Reported AP, RMOI for all runs, as well as aggregated information. Visual morphology is reported per run when available.

Peptide	Run	AP	RMOI	Mean AP	Mean RMOI	Is Fiber by RMOI (run)	Is Fiber by RMOI (agg)	Visual Morph Description (run)	Is Fiber Visually (run)	Is Fiber Visually (agg)
	run$_1$	2.889	0.758			✗		spherical	✗	
FPVSYAIY	run$_2$	2.902	0.731	2.931	0.757	✗	✗	spherical	✗	✗
	run$_3$	3.002	0.782			✗		spherical	✗	
	run$_1$	2.786	0.807			✗		spherical	✗	
PPFAQPMW	run$_2$	2.803	0.751	2.811	0.805	✗	✗	spherical	✗	✗
	run$_3$	2.842	0.856			✗		spherical	✗	
	run$_1$	2.749	0.830			✗		spherical	✗	
PFVCLIVF	run$_2$	2.777	0.703	2.771	0.806	✗	✗	spherical	✗	✗
	run$_3$	2.787	0.885			✗		spherical	✗	
	run$_1$	2.746	0.712			✗		spherical	✗	
PVFVIVVFP	run$_2$	2.765	0.845	2.766	0.799	✗	✗	spherical	✗	✗
	run$_3$	2.787	0.842			✗		spherical	✗	
	run$_1$	2.549	0.335			✓		fiber	✓	
PNVVYQVWYT	run$_2$	2.479	0.482	2.506	0.388	✗	✓	fiber	✓	✓
	run$_3$	2.490	0.348			✓		fiber	✓	
	run$_1$	2.480	0.794			✗		spherical	✗	
LFLIACTPY	run$_2$	2.529	0.257	2.503	0.524	✓	✗	fiber	✓	✗
	run$_3$	2.501	0.522			✗		spherical	✗	
	run$_1$	2.274	0.402			✗		fiber	✓	
WRWSDGCIL	run$_2$	2.233	0.495	2.256	0.435	✗	✗	wide fiber	✓	✓
	run$_3$	2.260	0.407			✗		disconnected fiber	✗	
	run$_1$	2.227	0.252			✓		fiber	✓	
VFESFTPY	run$_2$	2.266	0.332	2.221	0.282	✓	✓	fiber	✓	✓
	run$_3$	2.169	0.263			✓		fiber	✓	
	run$_1$	2.230	0.648			✗			✗	
WEFTGVNFT	run$_2$	2.190	0.510	2.209	0.539	✗	✗		✗	✗
	run$_3$	2.207	0.459			✗			✗	
LEYGFYCV	run$_1$	2.175	0.638	2.171	0.463	✗	✗	spherical with fiber-like parts	✗	✓
	run$_2$	2.159	0.339			✓		fiber	✓	
	run$_3$	2.178	0.414			✗		fiber	✓	
	run$_1$	2.097	0.306			✓		net-like aggregate	✗	
TLAGIRWVTE	run$_2$	2.023	0.499	2.058	0.436	✗	✗	net-like aggregate	✗	✗
	run$_3$	2.054	0.502			✗		fiber	✓	
	run$_1$	1.975	0.305			✓		fiber	✓	
FIRSESFL	run$_2$	1.987	0.286	1.961	0.352	✓	✓	fiber	✓	✓
	run$_3$	1.922	0.465			✗		fiber	✓	
	run$_1$	1.965	0.316			✓		fiber	✓	
IFCLIRSYLM	run$_2$	1.956	0.507	1.943	0.443	✗	✗	fiber	✓	✓
	run$_3$	1.909	0.506			✗		fiber	✓	
	run$_1$	1.697	0.336			✗			✗	
RLSHNAMLY	run$_2$	1.705	0.344	1.723	0.332	✗	✗		✗	✗
	run$_3$	1.766	0.317			✗			✗	
	run$_1$	1.549	0.543			✗			✗	
FVHIVNDQVV	run$_2$	1.579	0.419	1.551	0.521	✗	✗		✗	✗
	run$_3$	1.525	0.601			✗			✗	

Fibril-Targeted, PepMorph Candidates Cohort

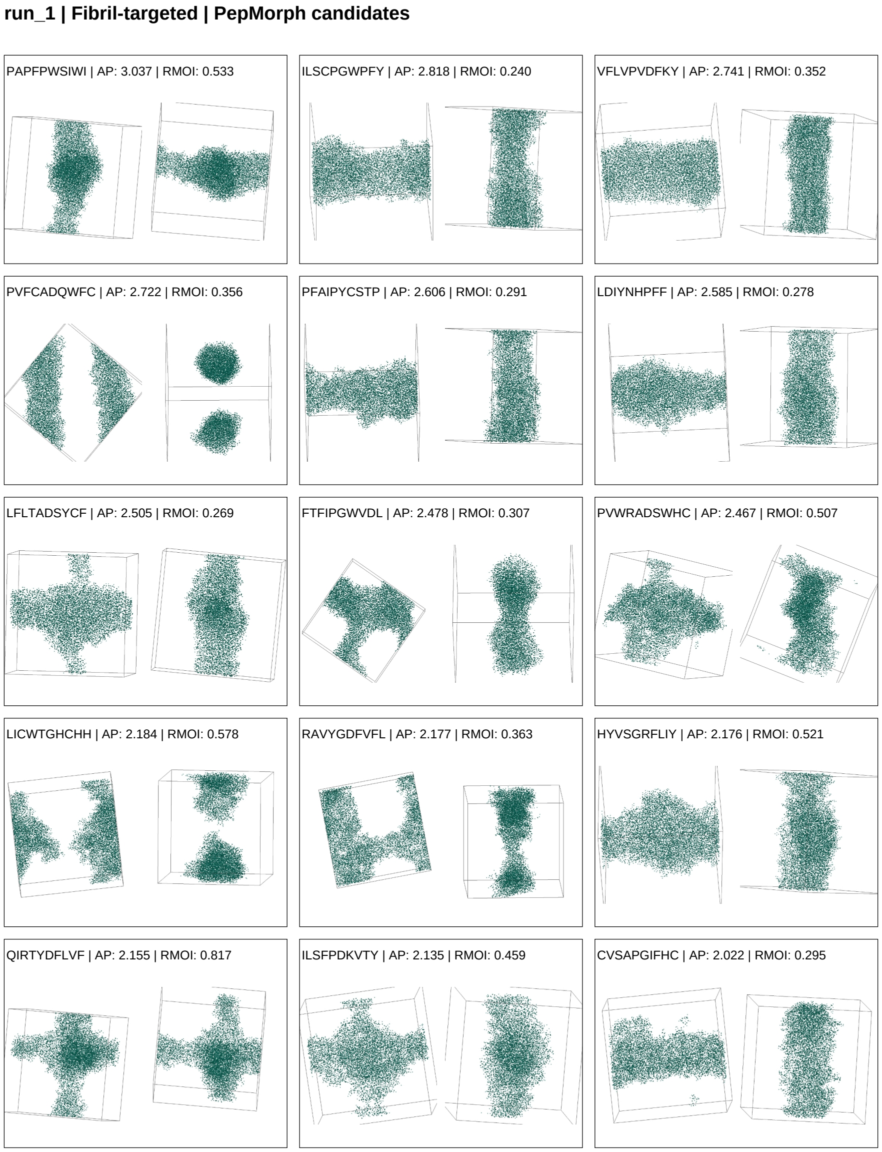

Figure 32: Visual representation, from two different views, of the final frame of the trajectory from the first run for the fibril-targeted PepMorph candidates, with corresponding AP and RMOI reported. Ordered from highest AP to lowest AP peptide in the cohort.

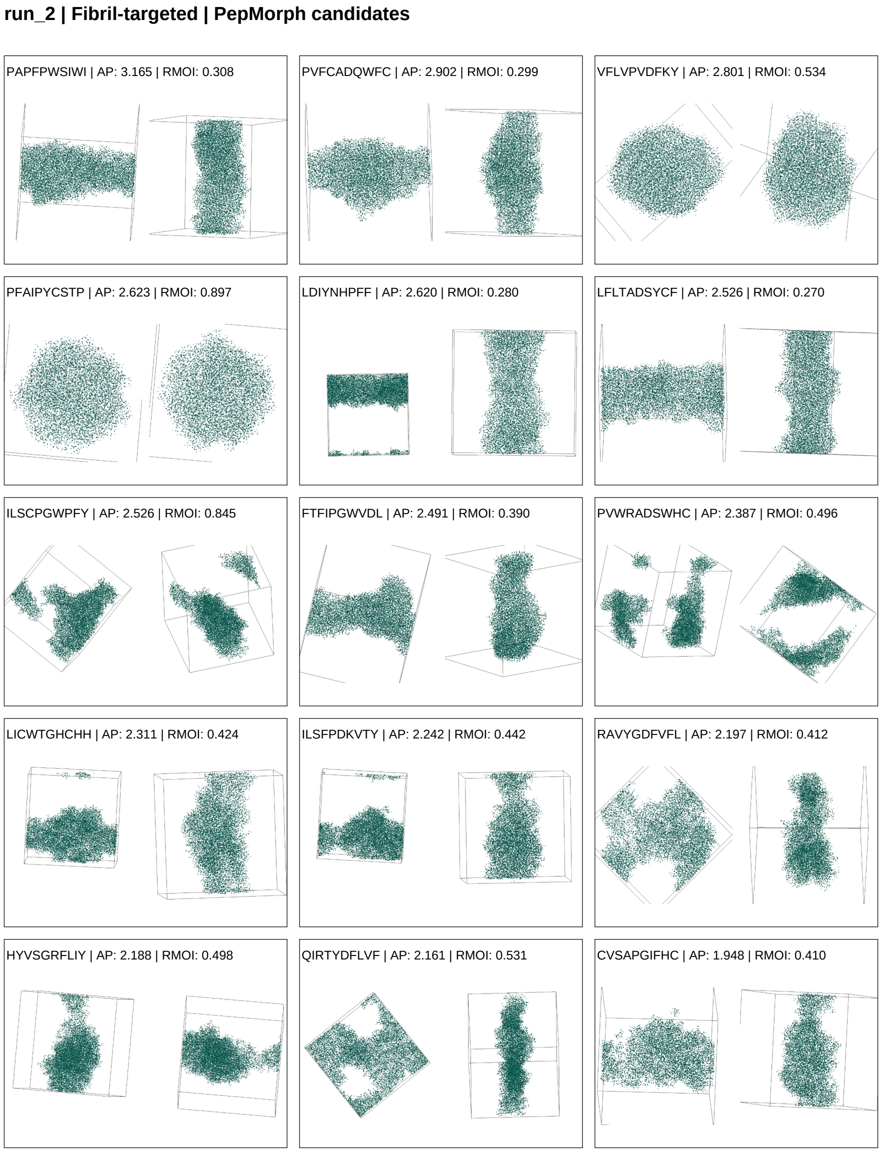

Figure 33: Visual representation, from two different views, of the final frame of the trajectory from the second run for the fibril-targeted PepMorph candidates, with corresponding AP and RMOI reported. Ordered from highest AP to lowest AP peptide in the cohort.

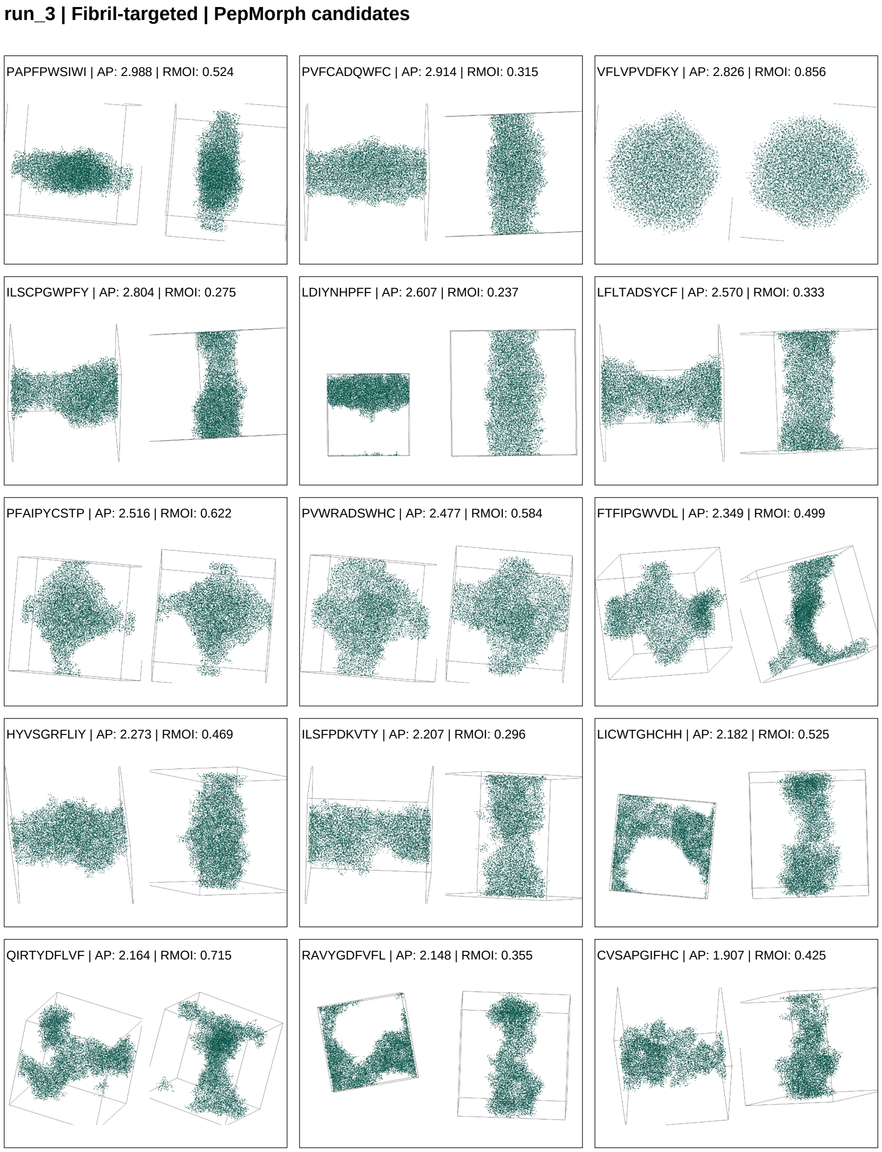

Figure 34: Visual representation, from two different views, of the final frame of the trajectory from the third run for the fibril-targeted PepMorph candidates, with corresponding AP and RMOI reported. Ordered from highest AP to lowest AP peptide in the cohort.

Table 12: Per-peptide results for target fibers. Reported AP, RMOI for all runs, as well as aggregated information. Visual morphology is reported per run when available.

Peptide	Run	AP	RMOI	Mean AP	Mean RMOI	Is Fiber by RMOI (run)	Is Fiber by RMOI (agg)	Visual Morph Description (run)	Is Fiber Visually (run)	Is Fiber Visually (agg)
	run$_1$	2.585	0.278			✓		Fiber	✓	
LDIYNHPFF	run$_2$	2.620	0.280	2.604	0.265	✓	✓	Fiber	✓	✓
	run$_3$	2.607	0.237			✓		Fiber	✓	
	run$_1$	2.505	0.269			✓		Fiber	✓	
LFLTADSYCF	run$_2$	2.526	0.270	2.533	0.291	✓	✓	Fiber	✓	✓
	run$_3$	2.570	0.333			✓		Fiber	✓	
	run$_1$	2.722	0.356			✗		Fiber	✓	
PVFCADQWFC	run$_2$	2.902	0.299	2.846	0.323	✓	✓	Fiber	✓	✓
	run$_3$	2.914	0.315			✓		Fiber	✓	
	run$_1$	2.177	0.363			✗		Intertwined Fiber	✓	
RAVYGDFVFL	run$_2$	2.197	0.412	2.174	0.377	✗	✗	Sheet String-Like	✗	✓
	run$_3$	2.148	0.355			✗		Intertwined Fiber	✓	
	run$_1$	2.022	0.295			✓		Fiber	✓	
CVSAPGIFHC	run$_2$	1.948	0.410	1.959	0.377	✗	✗	Fiber, Wide	✓	✓
	run$_3$	1.907	0.425			✗		Fiber, Narrow Center	✓	
	run$_1$	2.478	0.307			✓		Fiber	✓	
FTFIPGWVDL	run$_2$	2.491	0.390	2.439	0.399	✗	✗	Fiber	✓	✓
	run$_3$	2.349	0.499			✗		Fiber-Like, Helix Structure	✓	
ILSFPDKVTY	run$_1$	2.135	0.459	2.194	0.399	✗	✗	Fiber, connected along the box	✓	✓
	run$_2$	2.242	0.442			✗		Fiber, Wide	✓	
	run$_3$	2.207	0.296			✓		Fiber	✓	
	run$_1$	2.818	0.240			✓		Fiber	✓	
ILSCPGWPFY	run$_2$	2.526	0.845	2.716	0.453	✗	✓	Intertwined Fiber	✓	✓
	run$_3$	2.804	0.275			✓		Fiber	✓	
	run$_1$	3.037	0.533			✗		Intertwined Fiber	✓	
PAPFPWSIWI	run$_2$	3.165	0.308	3.063	0.455	✓	✗	Fiber	✓	✓
	run$_3$	2.988	0.524			✗		Intertwined Fiber	✓	
	run$_1$	2.176	0.521			✗		Fiber, Wide	✓	
HYVSGRFLIY	run$_2$	2.188	0.498	2.212	0.496	✗	✗	Net-Like	✗	✓
	run$_3$	2.273	0.469			✗		Fiber, Wide	✓	
LICWTGHCHH	run$_1$	2.184	0.578	2.226	0.509	✗	✗	Fiber, Connected In The Middle	✓	✓
	run$_2$	2.311	0.424			✗		Fiber, Wide	✓	
	run$_3$	2.182	0.525			✗		Curved Fiber	✓	
	run$_1$	2.467	0.507			✗		Intertwined Fiber	✓	
PVWRADSWHC	run$_2$	2.387	0.496	2.443	0.529	✗	✗	Two Fiber, Not Connected	✓	✓
	run$_3$	2.477	0.584			✗		Intertwined Fiber	✓	
	run$_1$	2.741	0.352			✗		Fiber	✓	
VFLVPVDFKY	run$_2$	2.801	0.534	2.789	0.581	✗	✗	Vesicle	✗	✗
	run$_3$	2.826	0.856			✗		Vesicle	✗	
	run$_1$	2.606	0.291			✓		Fiber	✓	
PFAIPYCSTP	run$_2$	2.623	0.897	2.582	0.603	✗	✗	Sphere	✗	✗
	run$_3$	2.516	0.622			✗		Split, Toward Spherical	✗	
QIRTYDFLVF	run$_1$	2.155	0.817	2.160	0.687	✗	✗	Two Fibers, Connected	✓	✓
	run$_2$	2.161	0.531			✗		Porous Sheet	✗	
	run$_3$	2.164	0.715			✗		Fiber	✓	

Robustness Controls: Box Size and Force-Field Sensitivity

To assess sensitivity of CG-MD outcomes to (i) periodic-boundary artifacts and (ii) force-field choice, we re-simulated a representative subset of validated peptides (8 per target morphology; 3 replicates each) under two protocol perturbations: (a) a larger cubic box with matched peptide concentration (24 nm; 1230 peptides), and (b) an alternative force field consistent with the AP sources used in dataset curation (MARTINI 2.2). All control runs used the same trajectory length, analysis pipeline, and morphology labeling criteria as the baseline simulations. Because the larger-box condition contains substantially more peptides at the same concentration, coarsening and morphology convergence may require longer trajectories; therefore, differences in fibrillar success rates can also be interpreted as protocol sensitivity and potentially slower convergence rather than a definitive change in thermodynamic preference.

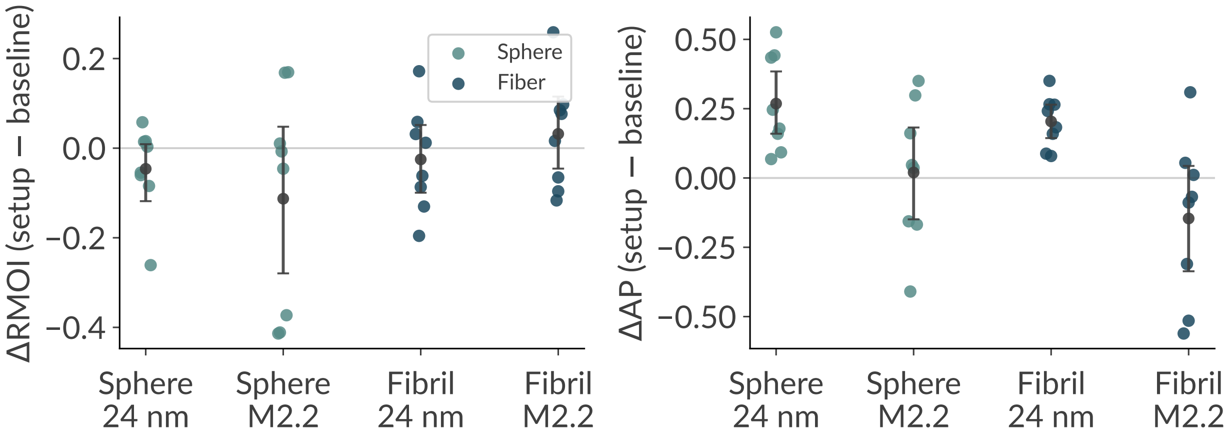

Figure 35: **Paired robustness controls relative to the baseline MD setup.** For each peptide (8 spheres, 8 fibrils; 3 replicates each), we compute the peptide-mean RMOI and AP in each setup and report paired differences relative to baseline (MARTINI 3, 15 nm): (a) ΔRMOI and (b) ΔAP for the larger-box condition (24 nm, concentration-matched) and MARTINI 2.2.

Table 13: **Robustness across MD protocol perturbations.** Morphology success (visual) is reported at the run level (24 runs per target and setup) and at the peptide level (majority vote over three runs; 8 peptides per target and setup) for the original setup (MARTINI 3, 15 nm box), the larger-box setup (MARTINI 3, 24 nm box), and a different force field (MARTINI 2.2, 15 nm box). Confidence intervals (CI) are Wilson 95% intervals on the corresponding success rates. We also report the mean and standard deviation (std.) of RMOI and aggregation propensity (AP) across runs.

Setup	Run success	Run CI	Peptide success	Peptide CI	RMOI (mean±std)	AP (mean±std)
**Spheres**						
MARTINI 3, 15 nm box	20/24 (83.3%)	[0.641, 0.933]	6/8 (75.0%)	[0.409, 0.929]	0.752 ± 0.170	2.436 ± 0.381
MARTINI 3, 24 nm box	19/24 (79.2%)	[0.595, 0.908]	7/8 (87.5%)	[0.529, 0.978]	0.705 ± 0.194	2.704 ± 0.545
MARTINI 2.2, 15 nm box	14/24 (58.3%)	[0.388, 0.755]	4/8 (50.0%)	[0.215, 0.785]	0.639 ± 0.192	2.455 ± 0.229
**Fibrils**						
MARTINI 3, 15 nm box	18/24 (75.0%)	[0.551, 0.880]	6/8 (75.0%)	[0.409, 0.929]	0.490 ± 0.186	2.341 ± 0.273
MARTINI 3, 24 nm box	10/24 (41.7%)	[0.245, 0.612]	4/8 (50.0%)	[0.215, 0.785]	0.465 ± 0.133	2.545 ± 0.343
MARTINI 2.2, 15 nm box	10/24 (41.7%)	[0.245, 0.612]	3/8 (37.5%)	[0.137, 0.694]	0.522 ± 0.095	2.195 ± 0.099

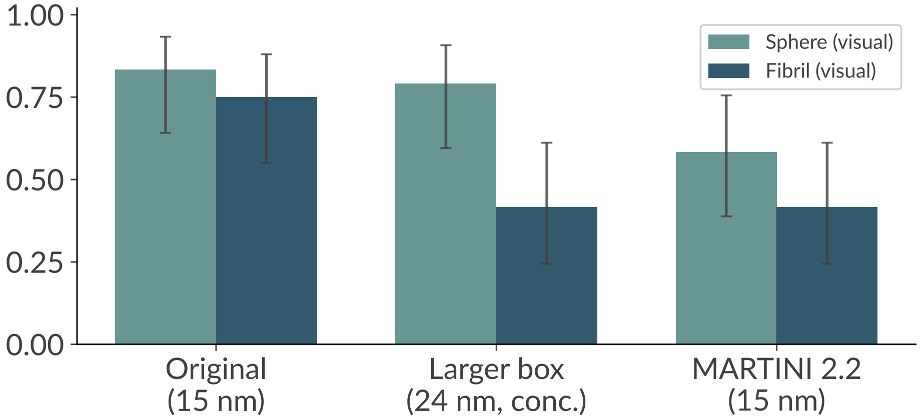

Figure 36: **Morphology success under MD protocol perturbations.** Run-level visual success rates (24 runs per target and setup) with Wilson 95% confidence intervals are shown for baseline (MARTINI 3, 15 nm), a larger box at matched concentration (24 nm; 1230 peptides), and MARTINI 2.2 (15 nm). Spherical outcomes remain broadly consistent under the larger-box condition, whereas fibrillar outcomes are more protocol-sensitive.

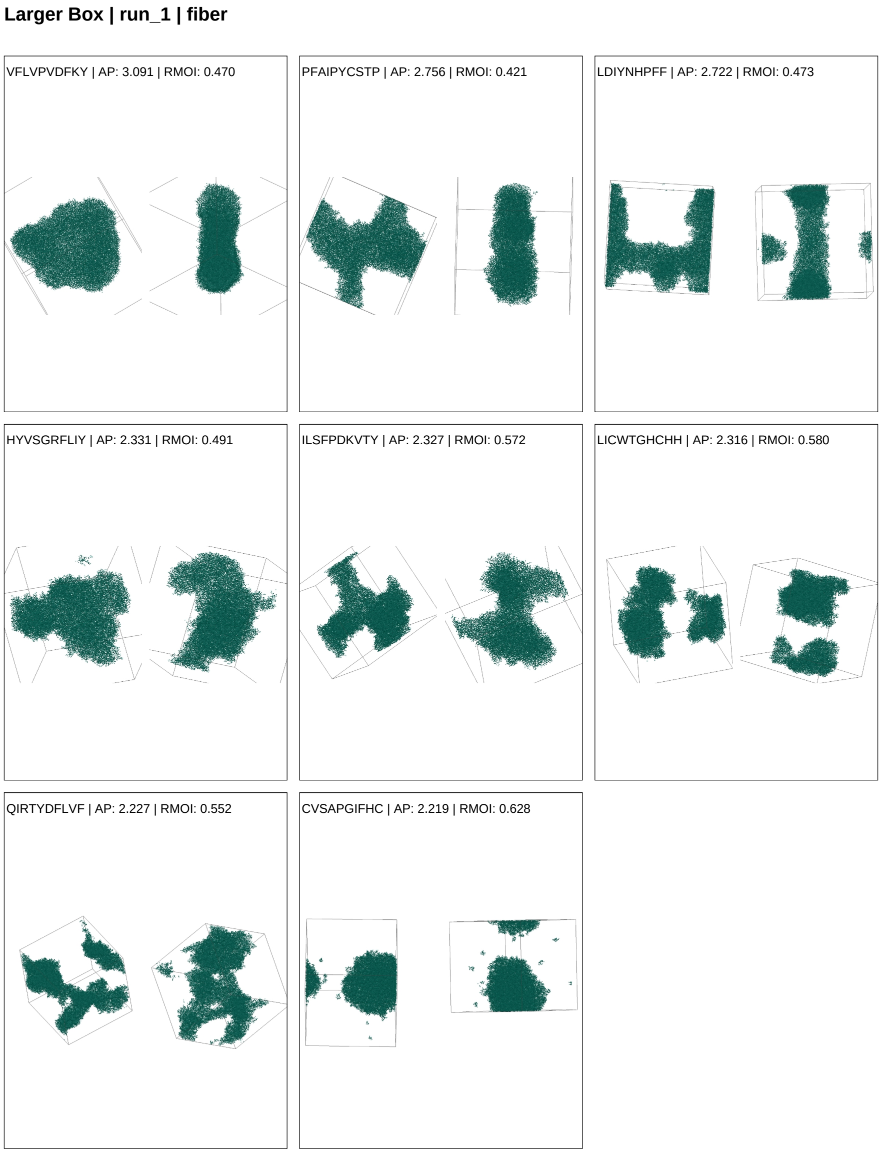

Figure 37: Visual representation, from two different views, of the final frame of the trajectory from the first run for the fibril-targeted candidates under the *Larger Box* control setup, with corresponding AP and RMOI reported. Ordered from highest AP to lowest AP peptide in the cohort.

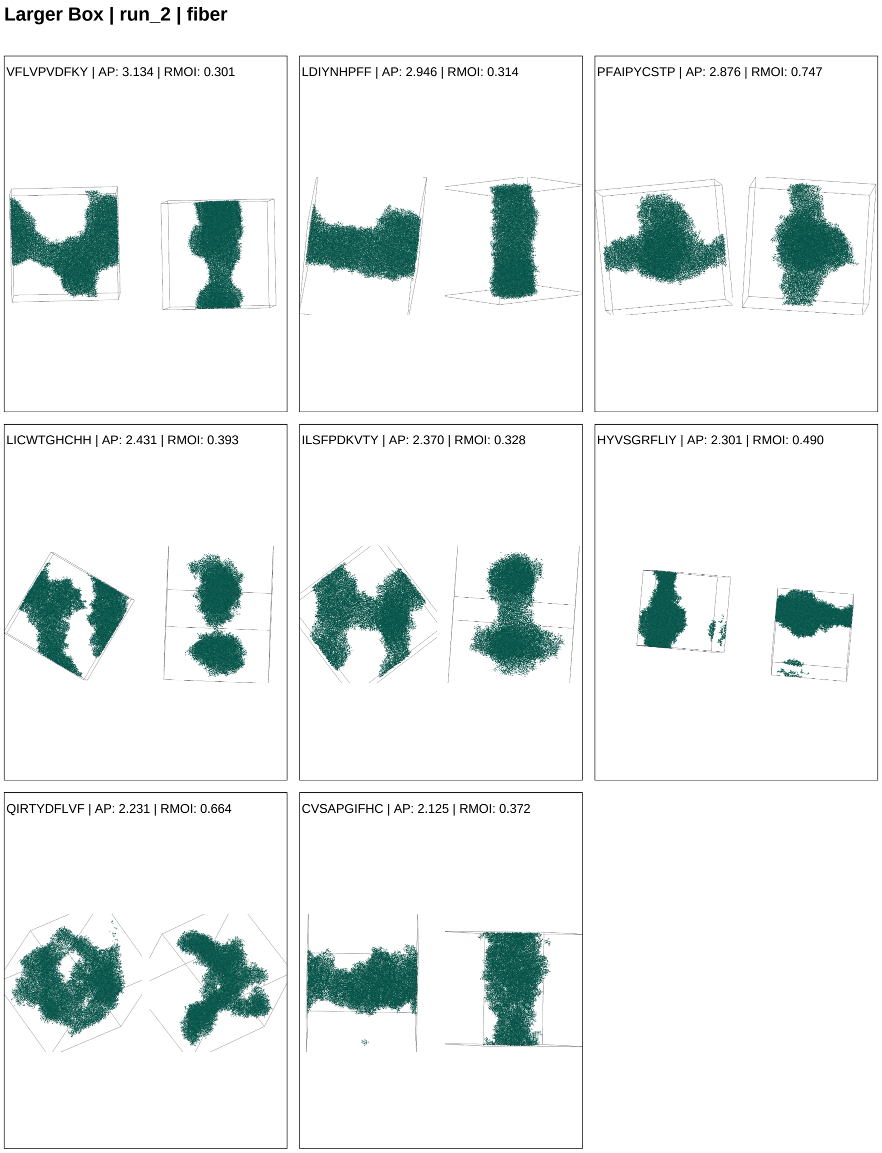

Figure 38: Visual representation, from two different views, of the final frame of the trajectory from the second run for the fibril-targeted candidates under the *Larger Box* control setup, with corresponding AP and RMOI reported. Ordered from highest AP to lowest AP peptide in the cohort.

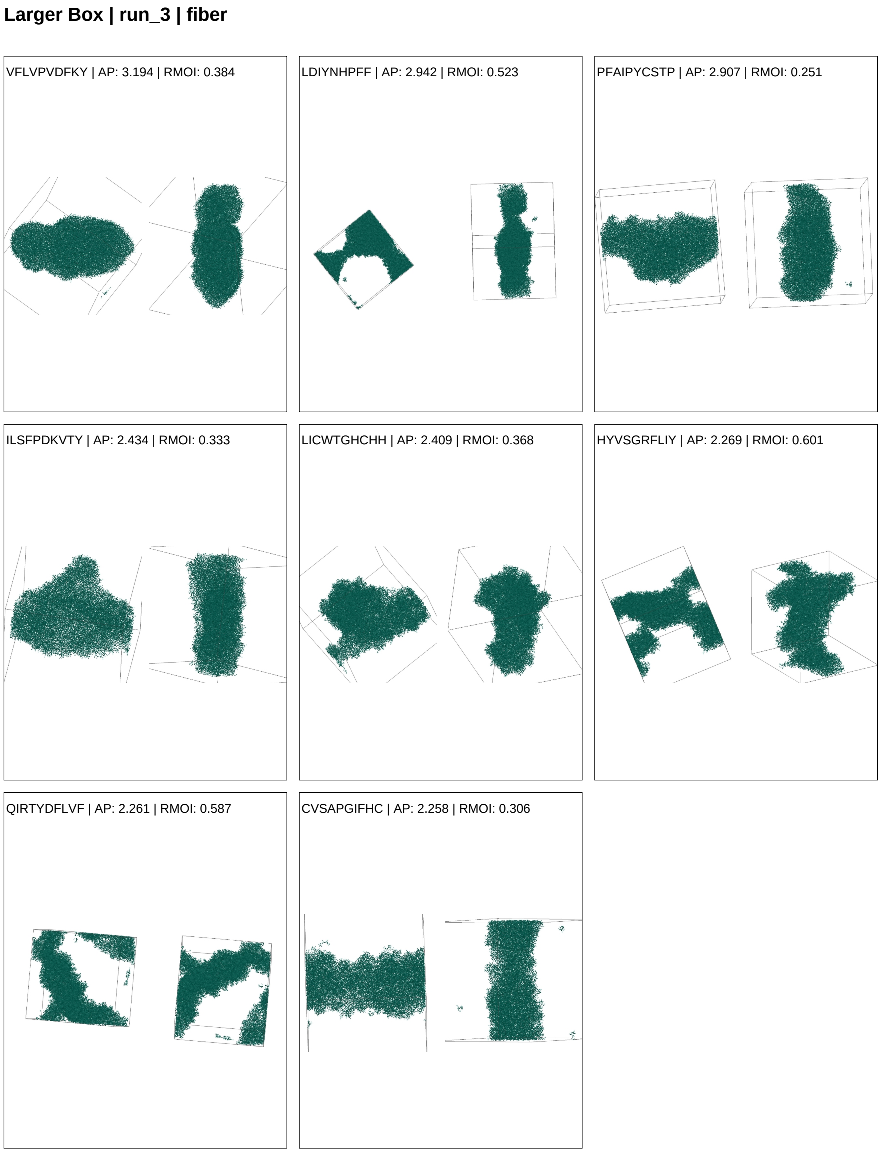

Figure 39: Visual representation, from two different views, of the final frame of the trajectory from the third run for the fibril-targeted candidates under the *Larger Box* control setup, with corresponding AP and RMOI reported. Ordered from highest AP to lowest AP peptide in the cohort.

Table 14: Per-peptide results for the larger-box fiber-targets robustness control (MARTINI 3, 24 nm box).

Peptide	Run	AP	RMOI	Mean AP	Mean RMOI	Is Fiber by RMOI (run)	Is Fiber by RMOI (agg)	Visual Morph Description (run)	Is Fiber Visually (run)	Is Fiber Visually (agg)
CVSAPGIFHC	run_1	2.219	0.628	2.201	0.435	✗	✗	wide spherical aggregate	✗	✓
	run_2	2.125	0.372			✗		fiber	✓	
	run_3	2.258	0.306			✓		fiber	✓	
	run_1	2.331	0.491			✗		irregular net	✗	
HYVSGRFLIY	run_2	2.301	0.490	2.300	0.528	✗	✗	irregular net	✗	✗
	run_3	2.269	0.601			✗		irregular net	✗	
	run_1	2.327	0.572			✗		net aggregate	✗	
ILSFPDKVTY	run_2	2.370	0.328	2.377	0.411	✓	✓	intertwined fiber	✓	✓
	run_3	2.434	0.333			✓		fiber	✓	
	run_1	2.722	0.473			✗		irregular net	✗	
LDIYNHPFF	run_2	2.946	0.314	2.870	0.437	✓	✗	amorphous	✗	✗
	run_3	2.942	0.523			✗		amorphous	✗	
LICWTGHCHH	run_1	2.316	0.580	2.385	0.447	✗	✗	one irregular fiber and a smaller amorphous	✓	✓
	run_2	2.431	0.393			✗		fiber	✓	
	run_3	2.409	0.368			✗		amorphous	✗	
	run_1	2.756	0.421			✗		fiber with a branch	✓	
PFAIPYCSTP	run_2	2.876	0.747	2.846	0.473	✗	✗	fibrillar aggregate with big spherical blob in the middle	✓	✓
	run_3	2.907	0.251			✓		fiber	✓	
	run_1	2.227	0.552			✗		net-like structure	✗	
QIRTYDFLVF	run_2	2.231	0.664	2.239	0.601	✗	✗	irregular net-like structure	✗	✗
	run_3	2.261	0.587			✗		irregular net-like structure	✗	
	run_1	3.091	0.470			✗		elongated vesicle	✗	
VFLVPVDFKY	run_2	3.134	0.301	3.140	0.385	✓	✗	curved irregular fiber	✓	✗
	run_3	3.194	0.384			✗		elongated vesicle	✗	

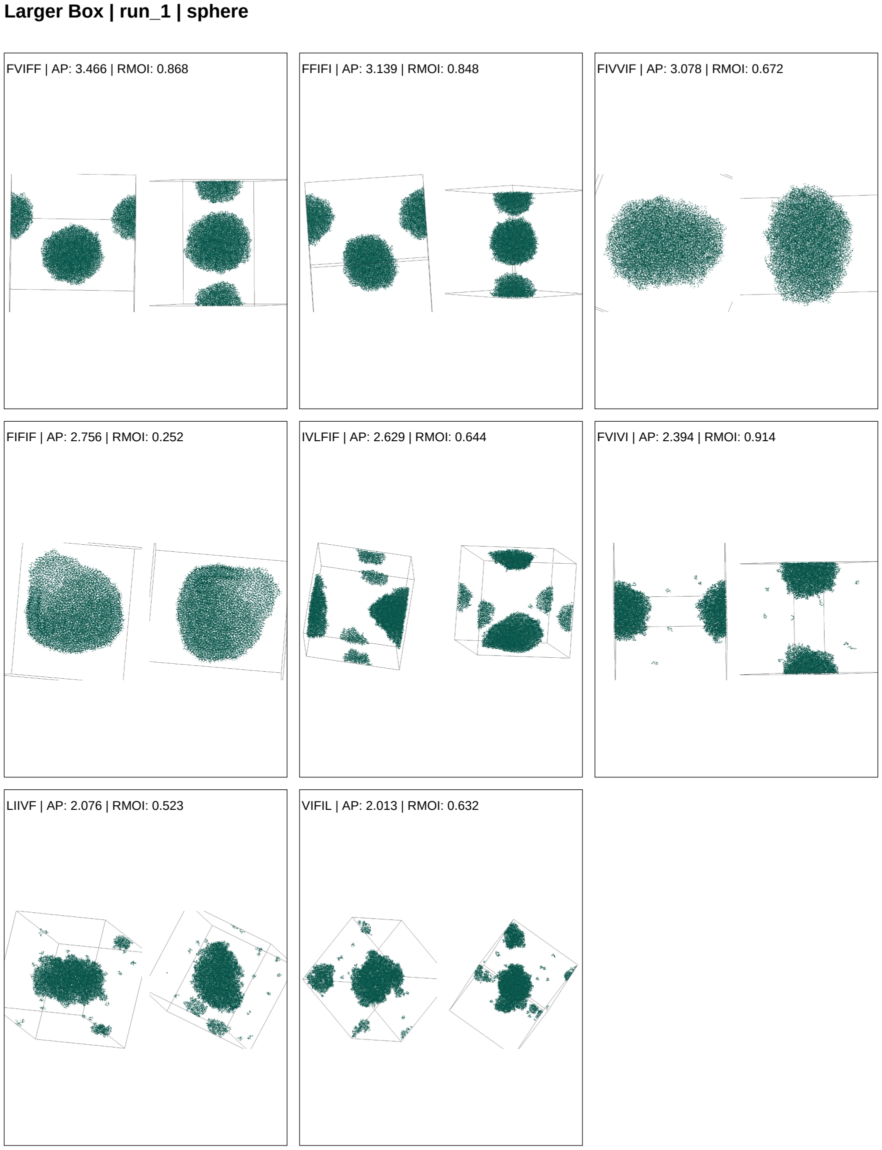

Figure 40: Visual representation, from two different views, of the final frame of the trajectory from the first run for the sphere-targeted candidates under the *Larger Box* control setup, with corresponding AP and RMOI reported. Ordered from highest AP to lowest AP peptide in the cohort.

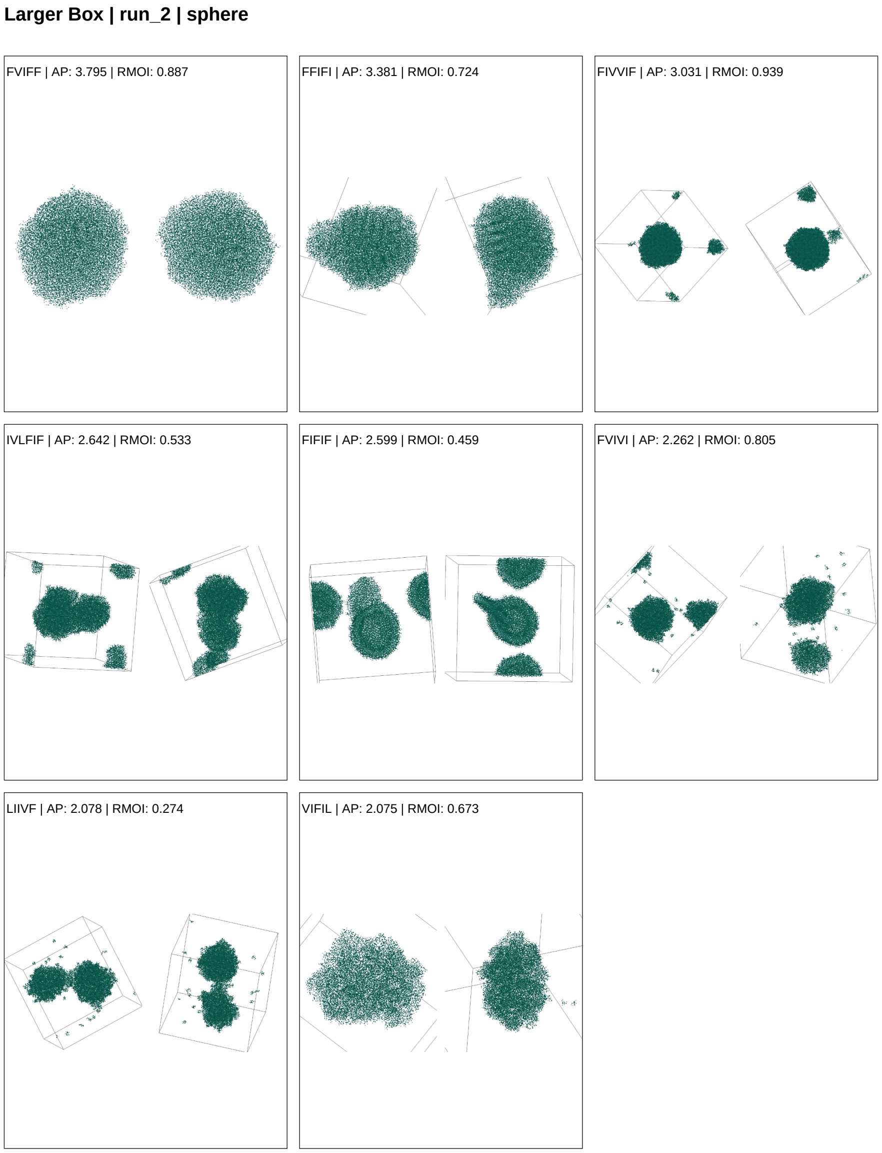

Figure 41: Visual representation, from two different views, of the final frame of the trajectory from the second run for the sphere-targeted candidates under the *Larger Box* control setup, with corresponding AP and RMOI reported. Ordered from highest AP to lowest AP peptide in the cohort.

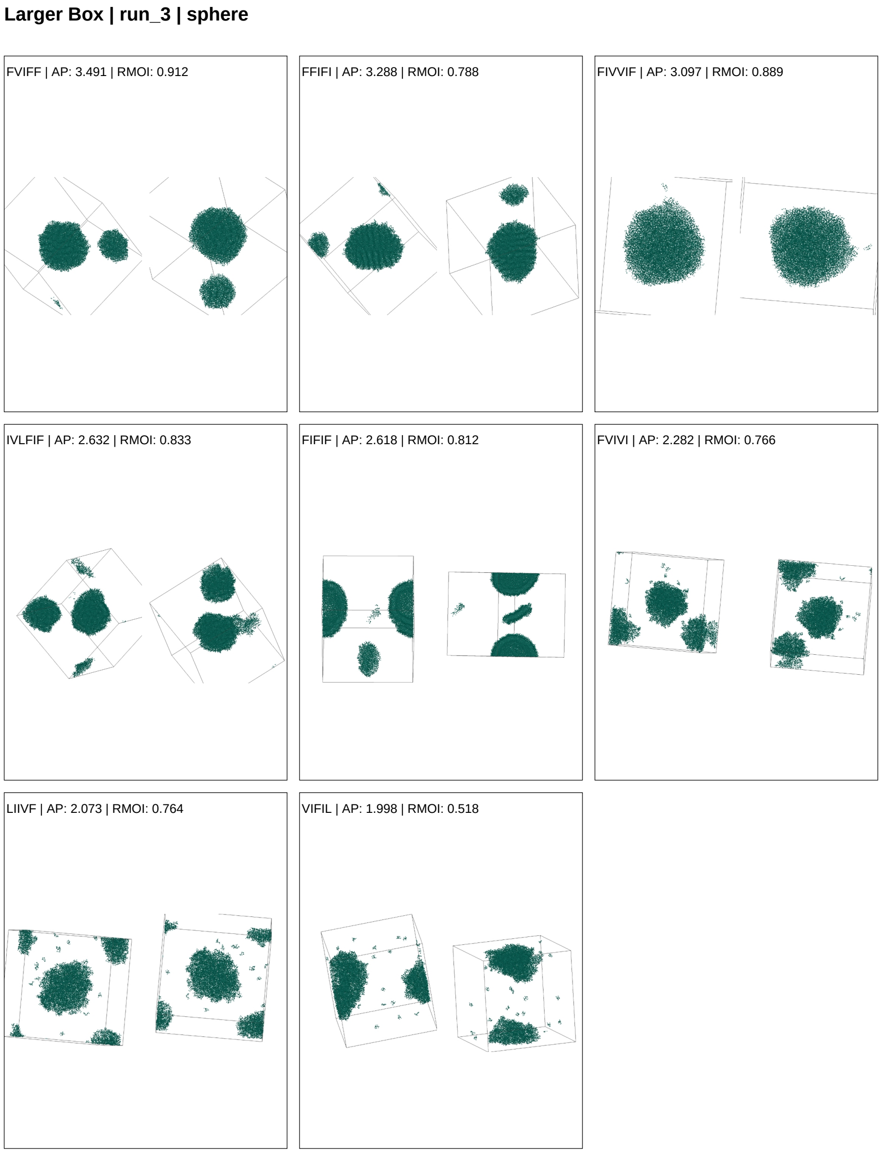

Figure 42: Visual representation, from two different views, of the final frame of the trajectory from the third run for the sphere-targeted candidates under the *Larger Box* control setup, with corresponding AP and RMOI reported. Ordered from highest AP to lowest AP peptide in the cohort.

Table 15: Per-peptide results for the larger-box sphere-targets robustness control (MARTINI 3, 24 nm box).

Peptide	Run	AP	RMOI	Mean AP	Mean RMOI	Is Sphere by RMOI (run)	Is Sphere by RMOI (agg)	Visual Morph Description (run)	Is Sphere Visually (run)	Is Sphere Visually (agg)
FFIFI	run_1	3.139	0.848	3.269	0.787	✓	✓	two spheres	✓	✓
	run_2	3.381	0.724			✗		one spherical aggregate with pi-pi stacking	✓	
	run_3	3.288	0.788			✓		one big spherical aggregate and a small one	✓	
FIFIF	run_1	2.756	0.252	2.657	0.508	✗	✗	squashed vesicle (spherical) aggregate disk-like	✗	✓
	run_2	2.599	0.459			✗		one vesicle and one amorphous, visibly not converged	✓	
	run_3	2.618	0.812			✓		clear vesicle (spherical)	✓	
FIVVIF	run_1	3.078	0.672	3.069	0.833	✗	✓	slightly elongated spherical aggregate	✓	✓
	run_2	3.031	0.939			✓		spherical aggregate	✓	
	run_3	3.097	0.889			✓		spherical aggregate	✓	
FVIFF	run_1	3.466	0.868	3.584	0.889	✓	✓	two same-sized spherical aggregates	✓	✓
	run_2	3.795	0.887			✓		one spherical aggregate	✓	
	run_3	3.491	0.912			✓		one large spherical aggregate and one small	✓	
FVIVI	run_1	2.394	0.914	2.313	0.829	✓	✓	spherical aggregate	✓	✓
	run_2	2.262	0.805			✓		one larger spherical aggregate and one smaller amorphous	✓	
	run_3	2.282	0.766			✓		two slightly irregular spherical aggregates	✓	
IVLFIF	run_1	2.629	0.644	2.634	0.670	✗	✗	iregular spherical blob	✓	✓
	run_2	2.642	0.533			✗		two elongated aggregates	✗	
	run_3	2.632	0.833			✓		two spheres	✓	
LIIVF	run_1	2.076	0.523	2.076	0.520	✗	✗	elongated blob aggregate	✗	✓
	run_2	2.078	0.274			✗		two spheres slightly connected	✓	
	run_3	2.073	0.764			✓		two spheres	✓	
VIFIL	run_1	2.013	0.632	2.029	0.608	✗	✗	two iregular spherical aggregates	✗	✗
	run_2	2.075	0.673			✗		approximately spherical aggregate	✓	
	run_3	1.998	0.518			✗		irregular approximately spherical aggregate	✗	

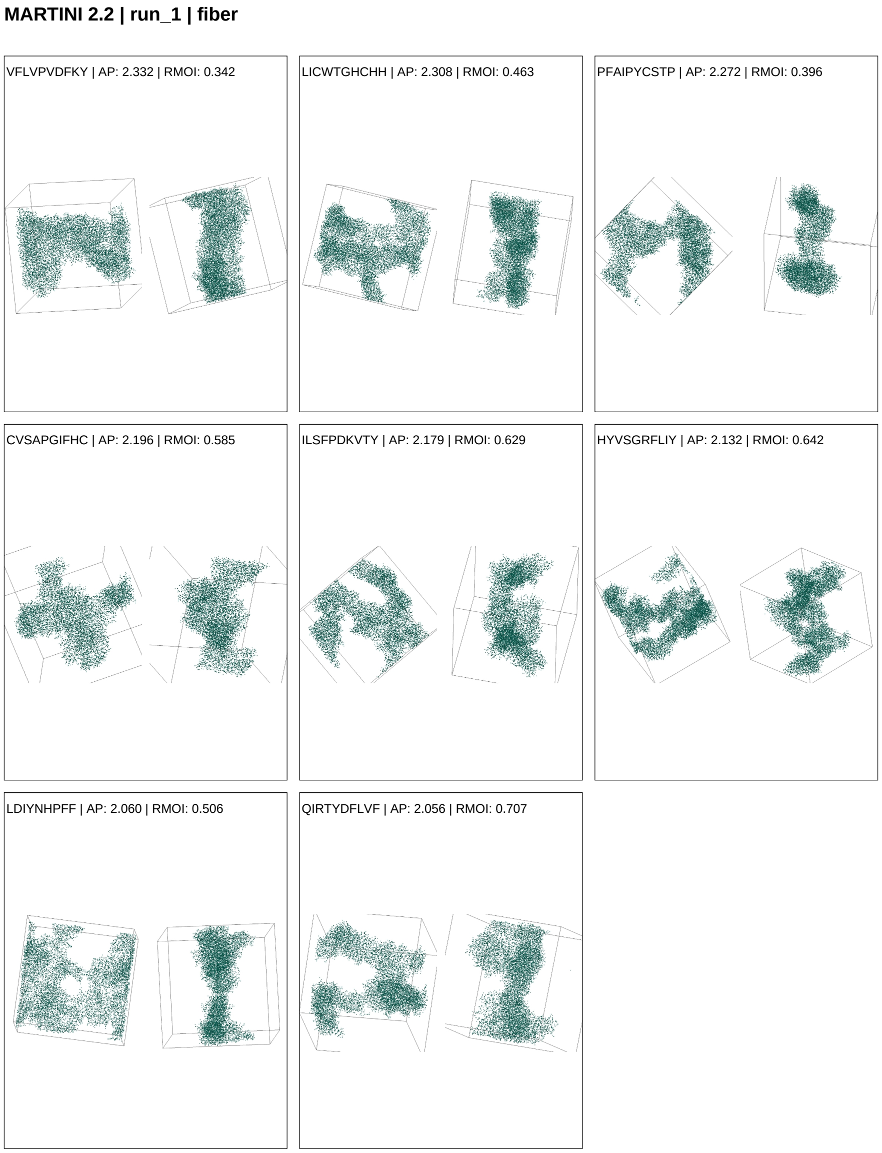

Figure 43: Visual representation, from two different views, of the final frame of the trajectory from the first run for the fibril-targeted candidates under the *MARTINI 2.2* control setup, with corresponding AP and RMOI reported. Ordered from highest AP to lowest AP peptide in the cohort.

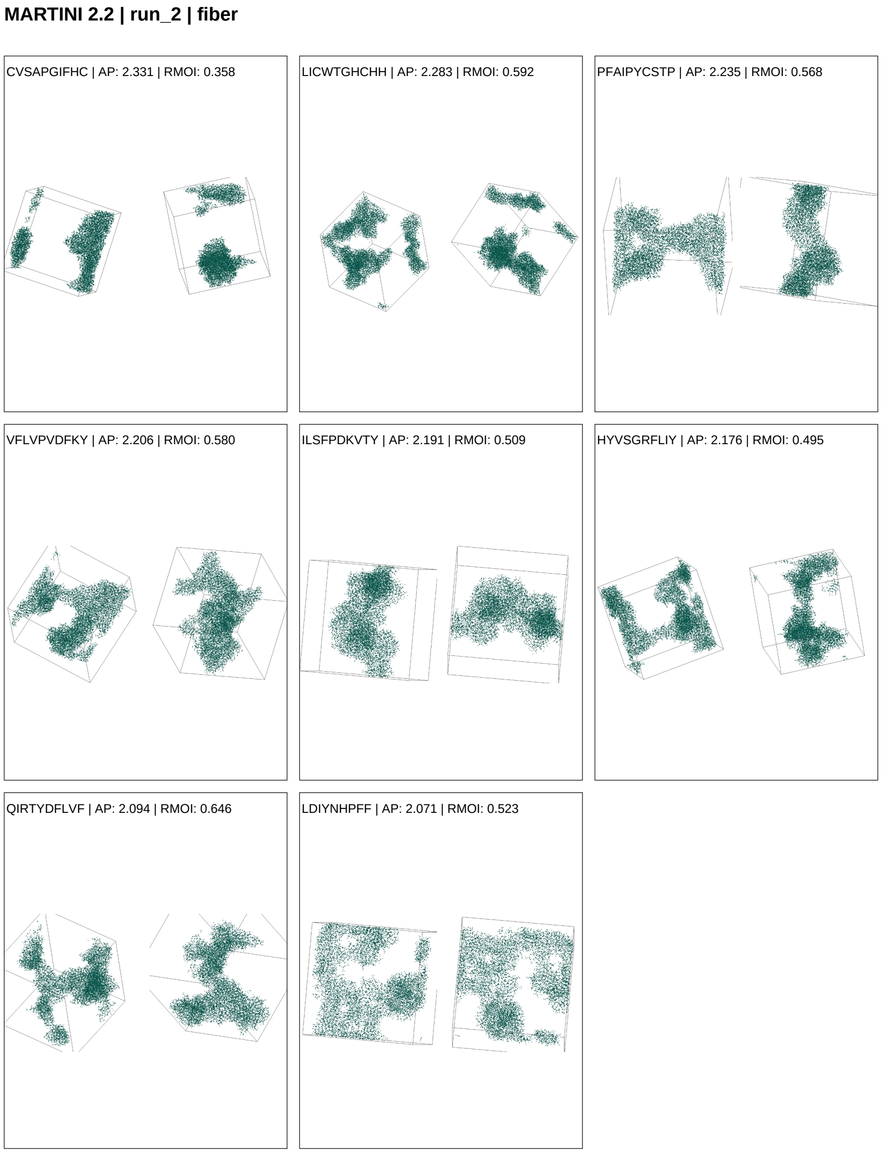

Figure 44: Visual representation, from two different views, of the final frame of the trajectory from the second run for the fibril-targeted candidates under the *MARTINI 2.2* control setup, with corresponding AP and RMOI reported. Ordered from highest AP to lowest AP peptide in the cohort.

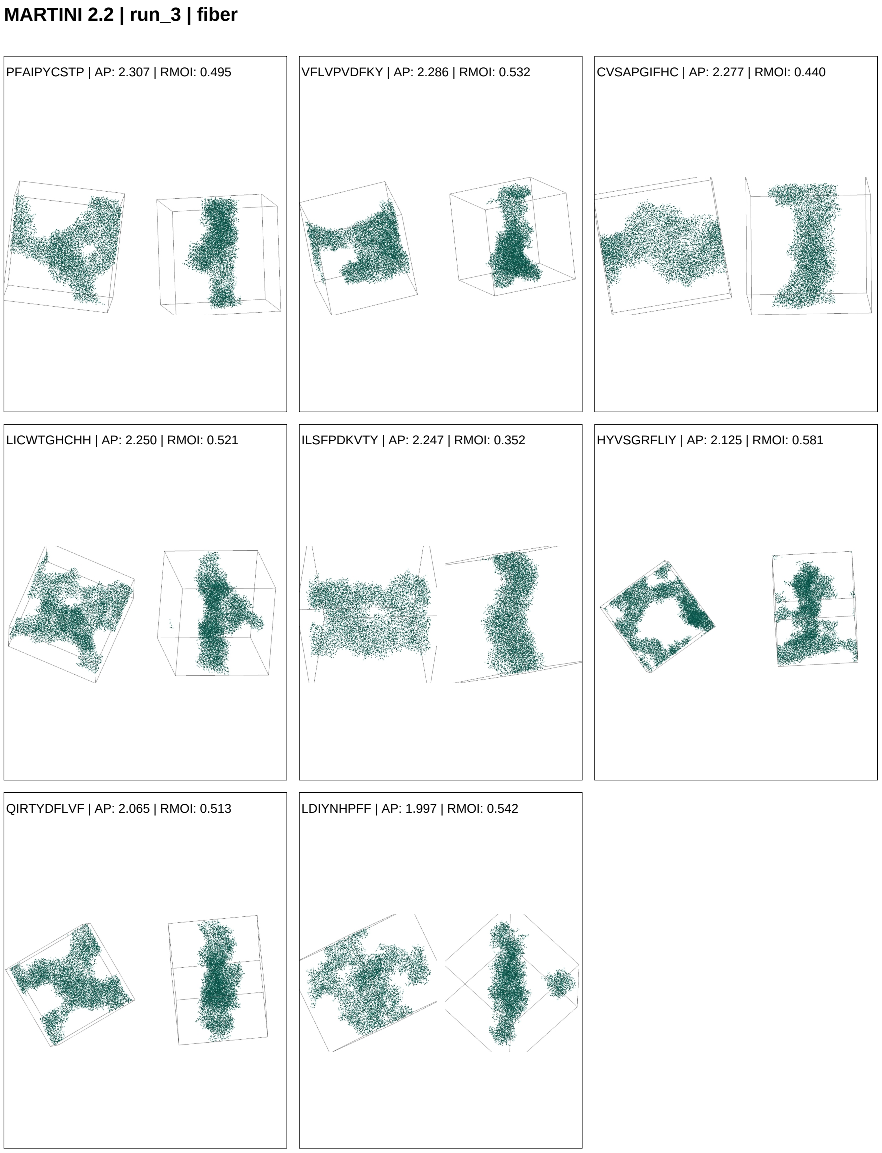

Figure 45: Visual representation, from two different views, of the final frame of the trajectory from the third run for the fibril-targeted candidates under the *MARTINI 2.2* control setup, with corresponding AP and RMOI reported. Ordered from highest AP to lowest AP peptide in the cohort.

Table 16: Per-peptide results for the alternative force-field fiber-targets robustness control (MARTINI 2.2, 15 nm box).

Peptide	Run	AP	RMOI	Mean AP	Mean RMOI	Is Fiber by RMOI (run)	Is Fiber by RMOI (agg)	Visual Morph Description (run)	Is Fiber Visually (run)	Is Fiber Visually (agg)
CVSAPGIFHC	run_1	2.196	0.585	2.268	0.461	✗	✗	intertwined fibers	✓	✓
	run_2	2.331	0.358			✗		fiber	✓	
	run_3	2.277	0.440			✗		wide fiber	✓	
HYVSGRFLIY	run_1	2.132	0.642	2.144	0.572	✗	✗	irregular net	✗	✗
	run_2	2.176	0.495			✗		irregular net	✗	
	run_3	2.125	0.581			✗		irregular net	✗	
ILSFPDKVTY	run_1	2.179	0.629	2.206	0.497	✗	✗	amorphous	✗	✗
	run_2	2.191	0.509			✗		irregular net	✗	
	run_3	2.247	0.352			✗		fiber-like structure	✓	
LDIYNHPFF	run_1	2.060	0.506	2.043	0.524	✗	✗	amorphous	✗	✗
	run_2	2.071	0.523			✗		amorphous	✗	
	run_3	1.997	0.542			✗		amorphous	✗	
LICWTGHCHH	run_1	2.308	0.463	2.280	0.525	✗	✗	fiber-like structure	✓	✓
	run_2	2.283	0.592			✗		one fiber-like structure and some aggregates almost connected	✓	
	run_3	2.250	0.521			✗		amorphous	✗	
PFAIPYCSTP	run_1	2.272	0.396	2.271	0.486	✗	✗	amorphous	✗	✓
	run_2	2.235	0.568			✗		intertwined fibers	✓	
	run_3	2.307	0.495			✗		intertwined fibers	✓	
QIRTYDFLVF	run_1	2.056	0.707	2.072	0.622	✗	✗	networked aggregation	✗	✗
	run_2	2.094	0.646			✗		networked aggregation	✗	
	run_3	2.065	0.513			✗		connected fibers	✓	
VFLVPVDFKY	run_1	2.332	0.342	2.274	0.484	✓	✗	irregular fiber	✓	✗
	run_2	2.206	0.580			✗		networked aggregation	✗	
	run_3	2.286	0.532			✗		elongated irregular aggregate	✗	

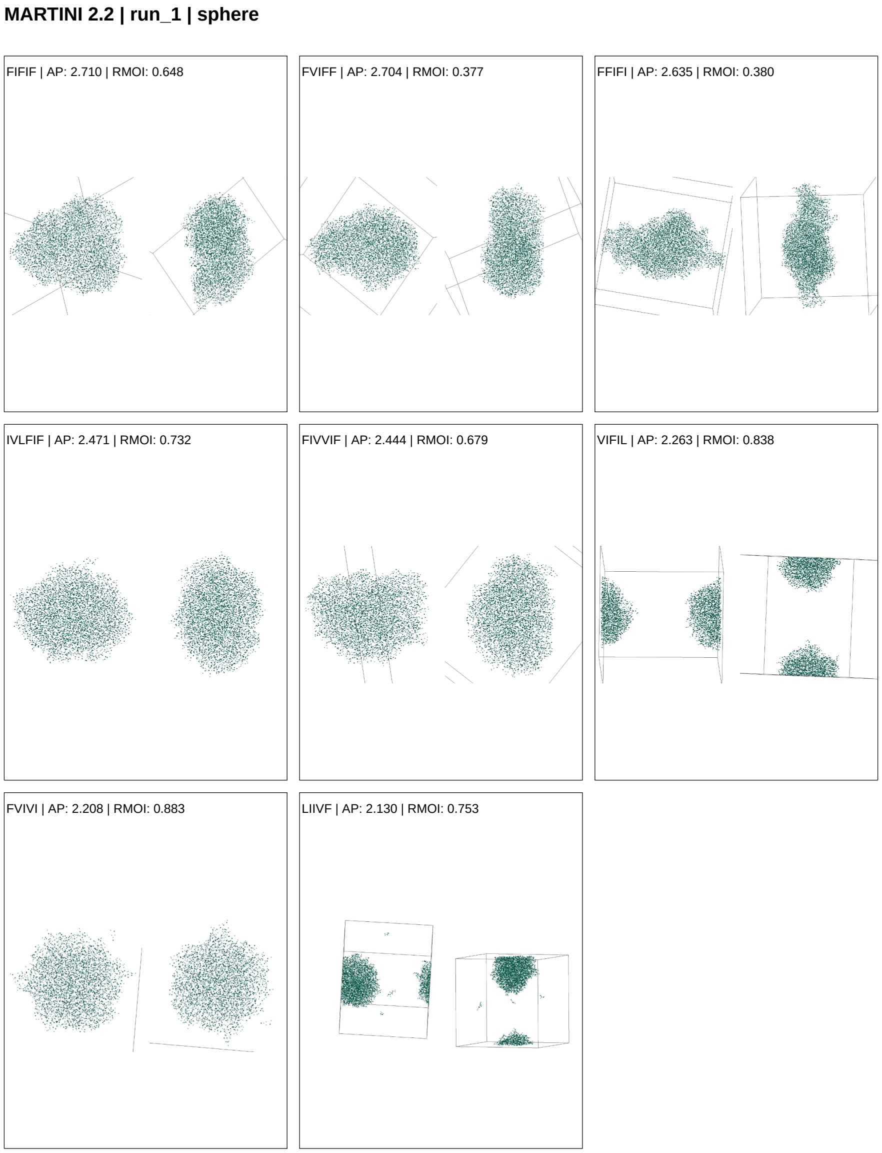

Figure 46: Visual representation, from two different views, of the final frame of the trajectory from the first run for the sphere-targeted candidates under the *MARTINI 2.2* control setup, with corresponding AP and RMOI reported. Ordered from highest AP to lowest AP peptide in the cohort.

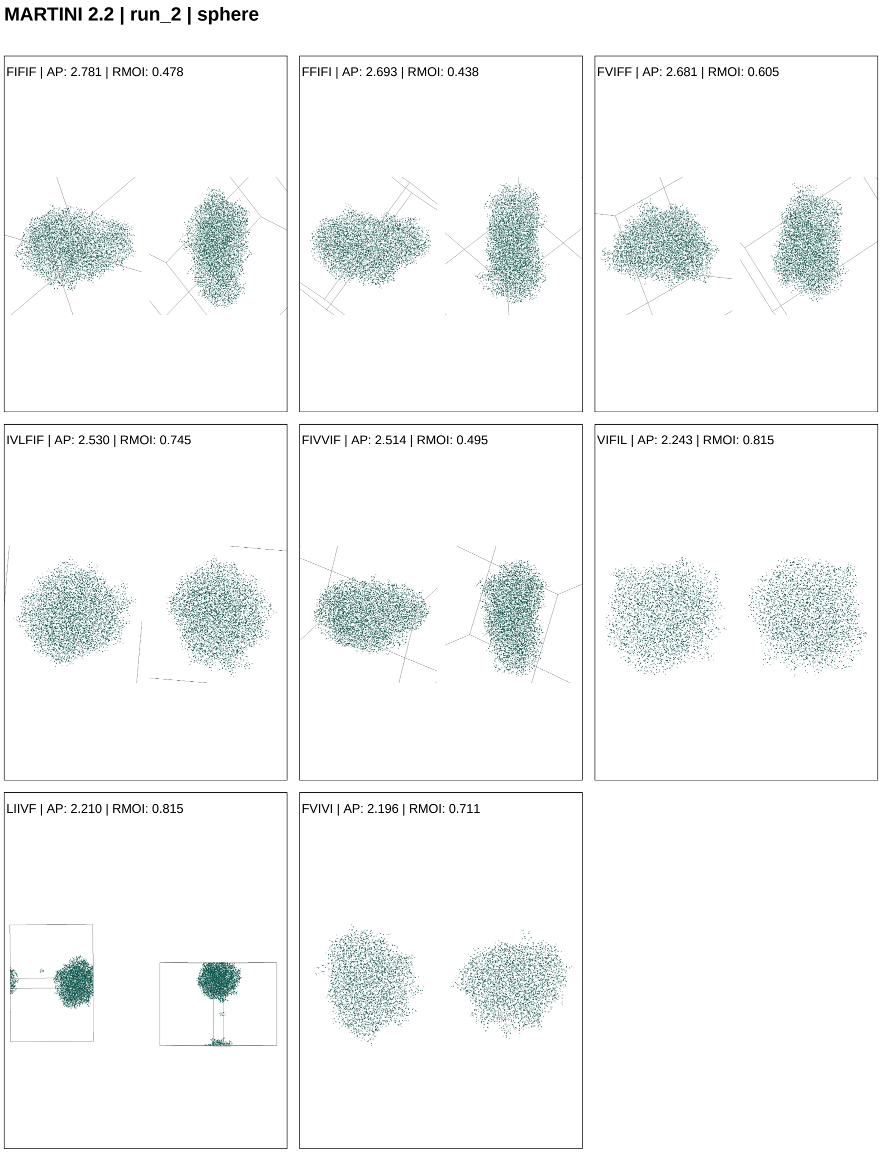

Figure 47: Visual representation, from two different views, of the final frame of the trajectory from the second run for the sphere-targeted candidates under the *MARTINI 2.2* control setup, with corresponding AP and RMOI reported. Ordered from highest AP to lowest AP peptide in the cohort.

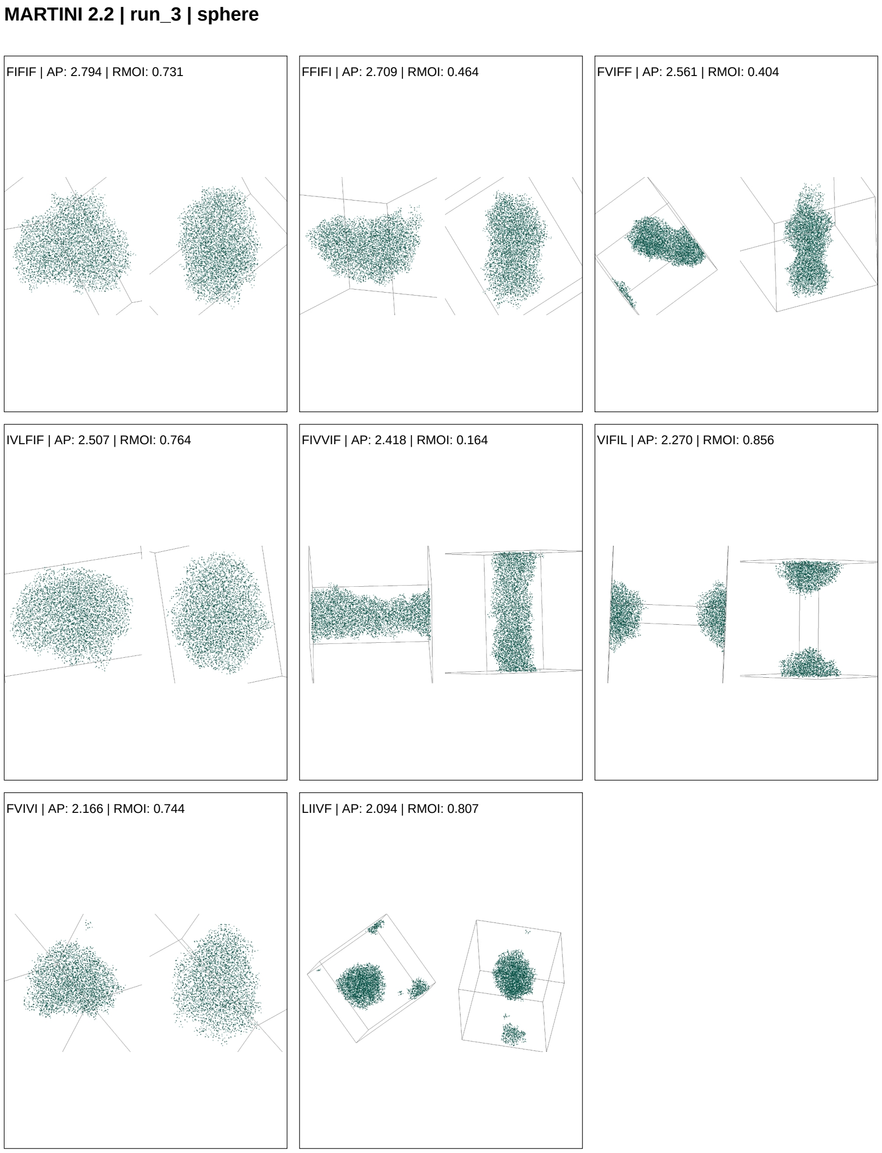

Figure 48: Visual representation, from two different views, of the final frame of the trajectory from the third run for the sphere-targeted candidates under the *MARTINI 2.2* control setup, with corresponding AP and RMOI reported. Ordered from highest AP to lowest AP peptide in the cohort.

Table 17: Per-peptide results for the alternative force-field sphere-targets robustness control (MARTINI 2.2, 15 nm box).

Peptide	Run	AP	RMOI	Mean AP	Mean RMOI	Is Sphere by RMOI (run)	Is Sphere by RMOI (agg)	Visual Morph Description (run)	Is Sphere Visually (run)	Is Sphere Visually (agg)
FFIFI	run_1	2.635	0.380	2.679	0.427	✗	✗	elongated aggregate almost disconnected	✗	✗
	run_2	2.693	0.438			✗		elongated spherical aggregate	✗	
	run_3	2.709	0.464			✗		elongated spherical aggregate	✗	
FIFIF	run_1	2.710	0.648	2.761	0.619	✗	✗	slightly elongated spherical aggregate	✗	✗
	run_2	2.781	0.478			✗		elongated aggregate	✗	
	run_3	2.794	0.731			✗		spherical aggregate	✓	
FIVVIF	run_1	2.444	0.679	2.458	0.446	✗	✗	slightly elongated spherical aggregate	✓	✗
	run_2	2.514	0.495			✗		elongated rod-like aggregate	✗	
	run_3	2.418	0.164			✗		rod aggregate	✗	
FVIFF	run_1	2.704	0.377	2.649	0.462	✗	✗	curved elongated aggregate	✗	✗
	run_2	2.681	0.605			✗		elongated aggregate	✗	
	run_3	2.561	0.404			✗		elongated aggregate	✗	
FVIVI	run_1	2.208	0.883	2.190	0.779	✓	✗	spherical aggregate	✓	✓
	run_2	2.196	0.711			✗		slightly elongated spherical aggregate	✓	
	run_3	2.166	0.744			✗		slightly squashed spherical aggregate	✓	
IVLFIF	run_1	2.471	0.732	2.503	0.747	✗	✗	spherical aggregate	✓	✓
	run_2	2.530	0.745			✗		spherical aggregate	✓	
	run_3	2.507	0.764			✓		spherical aggregate	✓	
LIIVF	run_1	2.130	0.753	2.145	0.792	✓	✓	spherical aggregate	✓	✓
	run_2	2.210	0.815			✓		spherical aggregate	✓	
	run_3	2.094	0.807			✓		spherical aggregate	✓	
VIFIL	run_1	2.263	0.838	2.259	0.837	✓	✓	spherical aggregate	✓	✓
	run_2	2.243	0.815			✓		spherical aggregate	✓	
	run_3	2.270	0.856			✓		spherical aggregate	✓	

Targeted fully atomistic re-simulations

The main-text morphology outcomes are defined under our CG-MD validation protocol and morphology criterion. To provide an orthogonal, higher-resolution sensitivity check, we additionally performed fully atomistic MD simulations for two sphere-target candidates (IVLFIF and VFFIF). These sequences are strongly hydrophobic and were therefore considered a priori plausible fiber-formers.

For each sequence, we ran one fully atomistic simulation and analyzed (i) aggregation propensity (AP) computed from peptide SASA as AP $= \overline{S}_{\text{first2}}/\overline{S}_{\text{last2}}$, where $\overline{S}_{\text{first2}} = (S_1 + S_2)/2$ and $\overline{S}_{\text{last2}} = (S_{T-1} + S_T)/2$; and (ii) an RMOI time-series of the largest cluster to quantify compactness versus anisotropy. Both sequences aggregate strongly (AP > 3) but yield anisotropic, fiber-like assemblies with intermediate RMOI values (Fig. 49).

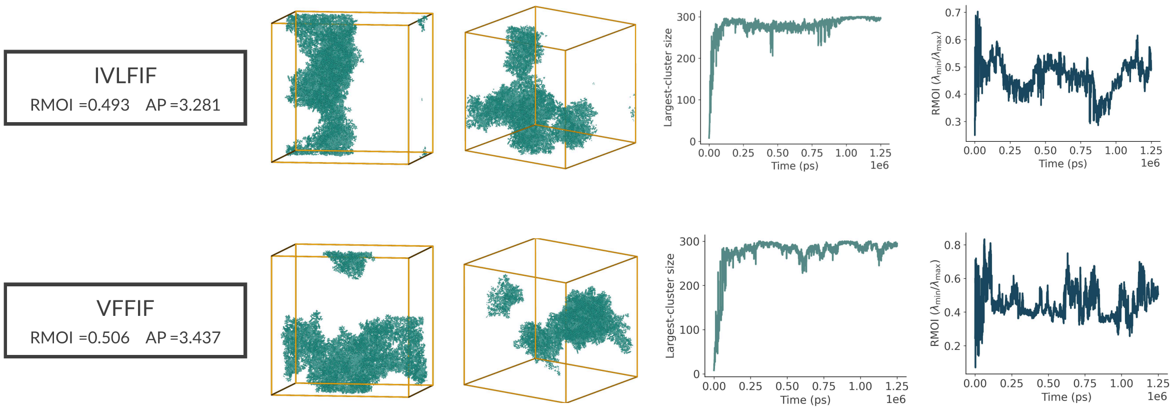

Figure 49: **Atomistic simulation summary.** From left to right, we report the RMOI and AP for IVLFIF and VFFIF, followed by last-frame snapshots of the atomistic simulations, and time series of the largest-cluster size and RMOI.

These targeted atomistic results support that morphology can be resolution- and protocol-dependent, especially for short, highly hydrophobic peptides. Accordingly, we interpret the main-text morphology success rate as conditional on the CG-MD protocol, and we treat monomer- or proxy-based conditioning as heuristic steering rather than a deterministic morphology guarantee. In future work, adding expert-driven constraints (e.g., an explicit hydrophobicity cap for sphere targeting) may further improve robustness without changing the

core pipeline.
